# Keratin23 (KRT23) Knockdown Decreases Proliferation and Affects the DNA Damage Response of Colon Cancer Cells

**DOI:** 10.1371/journal.pone.0073593

**Published:** 2013-09-09

**Authors:** Karin Birkenkamp-Demtröder, Stephan A. Hahn, Francisco Mansilla, Kasper Thorsen, Abdelouahid Maghnouj, Rikke Christensen, Bodil Øster, Torben Falck Ørntoft

**Affiliations:** 1 Department of Molecular Medicine MOMA, Aarhus University Hospital, Skejby, Aarhus N, Denmark; 2 Department of Molecular GI-Oncology, Center of Clinical Research, Ruhr-University Bochum, Bochum, Germany; 3 Department of Clinical Genetics, Aarhus University Hospital, Skejby, Aarhus N, Denmark; University Medical Center Hamburg-Eppendorf, Germany

## Abstract

Keratin 23 (KRT23) is strongly expressed in colon adenocarcinomas but absent in normal colon mucosa. Array based methylation profiling of 40 colon samples showed that the promoter of KRT23 was methylated in normal colon mucosa, while hypomethylated in most adenocarcinomas. Promoter methylation correlated with absent expression, while increased KRT23 expression in tumor samples correlated with promoter hypomethylation, as confirmed by bisulfite sequencing. Demethylation induced KRT23 expression *in vitro.* Expression profiling of shRNA mediated stable KRT23 knockdown in colon cancer cell lines showed that KRT23 depletion affected molecules of the cell cycle and DNA replication, recombination and repair. *In vitro* analyses confirmed that KRT23 depletion significantly decreased the cellular proliferation of SW948 and LS1034 cells and markedly decreased the expression of genes involved in DNA damage response, mainly molecules of the double strand break repair homologous recombination pathway. KRT23 knockdown decreased the transcript and protein expression of key molecules as e.g. MRE11A, E2F1, RAD51 and BRCA1. Knockdown of KRT23 rendered colon cancer cells more sensitive to irradiation and reduced proliferation of the KRT23 depleted cells compared to irradiated control cells.

## Introduction

Colorectal cancer (CRC) accounts for approximately 10% of the total worldwide cancer cases with an overall five years survival of around 50% [1]. Early diagnosis and better treatment of CRC requires the identification of new biomarkers as well as insights into the molecular mechanisms of colorectal carcinogenesis.

Two major molecular subgroups of colon cancer exist, microsatellite instable (MSI) and microsatellite stable (MSS) [2], where MSI tumors represent approximately 15% of the total incidence [3]. Microsatellite instable tumors show mutations or epigenetic alterations in the mismatch repair genes that lead to alterations in microsatellite DNA (short repeated sequences of DNA). Increasing evidence suggests that MSI tumors are associated with better prognosis [4] and that patients with MSI may not benefit from fluorouracil-based adjuvant chemotherapy [5] [6].

Several epigenetic abnormalities have been described for CRC [7]. Aberrant methylation in the colon can be observed already in early premalignant lesions as well as in tumor-adjacent normal-appearing mucosa. Epigenetic gene activation based on DNA demethylation or hypomethylation of the promoter region is involved in the initiation and progression of cancer [7].

Keratins are the intermediate filament forming proteins of epithelial cells. Today, 54 mammalian keratins are known, 28 type I (acidic) and 26 type II (basic-to-neutral) keratins [8]. Several studies have provided evidence for active keratin involvement in cancer cell proliferation, invasion and metastasis, as well as in treatment responsiveness. Furthermore, it has been suggested to further explore the role of keratins as multifunctional regulators of epithelial tumorigenesis [9].

Keratin 23 (*KRT23*) belongs to the acidic type I keratins [10]. The KRT23 transcript was found to be highly induced in the human pancreatic cancer cell line AsPC-1 upon treatment with a histone deacetylase inhibitor (HDACi) [11]. Further, K23 was identified as a tumor-associated antigen in sera from patients with hepatocellular carcinoma [12]. In previous studies, we identified the KRT23 transcript and K23 protein to be strongly upregulated in colon adenocarcinomas compared to normal mucosa [13] [14]. Neither the KRT23 transcript nor the K23 protein has been shown to be a prognostic marker in colon cancer; however, we observed a tendency that patients with stage II colon adenocarcinomas with high KRT23 transcript levels seemed to have a lower recurrence rate. The phosphoprotein K23 accumulated in the Golgi of the majority of MSS tumors, while most of the MSI tumors showed minor cytoplasmic expression or were completely negative for K23 protein expression [14]. Overexpression of KRT23 reduced the cellular viability of MSI cell lines while it had no significant functional impact on the MSS cell lines [14]. Promoter binding analysis identified several genes correlating with KRT23 expression in adenocarcinomas. Recently, the smad4 inducible K23 protein was identified to interact with keratins K8 and K18, heat shock proteins 60 and 70, plectin 1, as well as 14-3-3epsilon and gamma [15], suggesting an important role for K23 in regulatory processes. Its regulatory role may also be supported by very recent findings by Starman et al identifying KRT23 as a potential biomarker for steatohepatitis [16].

The aim of this study was to identify a regulatory mechanism for KRT23 expression. Furthermore, we intended to identify downstream target genes and associated pathways upon KRT23 knockdown, to elucidate the impact of KRT23 depletion on the molecular and cellular functions of cancer cells.

## Materials and Methods

Informed written consent was obtained from all patients and all studies were approved by the Central Denmark Region Committees on Biomedical Research Ethics.

### Whole Genome Methylation Analysis

Genomic DNA from serial cryosections was extracted using Puregene DNA purification kit (Gentra Systems, Plymouth, MN). When necessary, tumor biopsies were macroscopically trimmed to enrich the fraction of neoplastic cells to a minimum of 60% prior to DNA isolation. Median cancer cell percentage was 80%. One microgram of DNA was bisulfite modified using EpiTect Bisulfite Kit (Qiagen, Copenhagen, Denmark) using EZ-96 DNA Methylation D5004 (Zymo Research, Orange, CA) for microarrays and bisulfite sequencing. Bisulfite modified DNA was whole genome amplified and hybridized to Infinium HumanMethylation27 BeadChips (Illumina, San Diego, CA) overnight as described by the manufacturer. BeadChips were scanned with a BeadXpress Reader instrument (Illumina) and data analyzed using Bead Studio Methylation Module Software (Illumina) as described in detail in [17]. Methylation levels were provided in beta values, with a beta value of 0 corresponding to no methylation, and 1 corresponding to full methylation. For comparison of methylation status versus expression data, log2 expression intensities were obtained by microarray expression profiling of the same samples, samples were normalized by RMA and transcript intensities log2<5 were regarded as “not expressed”.

### Bisulfite Sequencing

Bisulfite modified DNA was amplified using primers designed with MethPrimer (http://www.urogene.org/methprimer/index1.html). PCR products were gel-purified and cloned using TOPO TA Cloning ® Kit for Sequencing (Invitrogen,Taastrup, Denmark). PCR amplification for sequencing was performed directly on the colonies with M13 primers (DNA Technology, Risskov, Denmark) and TEMPase DNA Polymerase (Amplicon, Skovlunde, Denmark). For each gene, 10 clones were randomly selected and sequenced using BigDye terminator cycle sequencing kit and a 3130xl Genetic Analyzer (Applied Biosystems, Foster City, CA). In detail, we analyzed a sequence comprising 253 bp overlapping the transcription start (+1) and the first KRT23 exon. Genomic DNA was isolated from two different sets, 23 tumor biopsy patient samples (4 Normal mucosa, 3 adenomas, 5 MSI and 12 MSS) and the cells AZA treated HCT116 cells described above using the GENTRA PUREGENE DNA Purification Kit (Qiagen). DNA was bisulfite converted using the MethylEasy DNA Bisulfite Modification Kit (Diagenode).

The 253 bp KRT23 amplicon was PCR amplified with TEMPase Hot Start DNA Polymerase (Amplicon) using a mix containing primers F1(C): 5′– GTGGTTTTCGTTTTTAGATTGTTT-3′ and F1(T) 5′- GTGGTTTTTGTTTTTAGATTGTTT and R1∶5′- TCAAAACCAAACAACCCTAACCTA-3′. The amplicons were gel purified (Gel 11Band Purification Kit; GE Healthcare) and subcloned into the pCR4-TOPO vector (Invitrogen) were 12–16 clones from each experiment were sequenced using M13 forward primers. For visualization of methylation status, we used the following software: http://quma.cdb.riken.jp/.

### Colon Cell Lines

Obtained from American Type Culture Collection (ATCC-LGC standards, Borås, Sweden) or obtained from the Hahn lab were re-authenticated via STR analysis [18] using the Cell-ID-system (G9500, Promega, Nacka, Sweden), products were analyzed on an Applied-Biosystems3130 Genetic Analyzer. No mycoplasma contamination was detected using nested PCR-based mycoplasma detection. Colon cancer cell lines in this study were HCT116 (MSI), DLD1 (MSI), SW480 (MSS, p53 mutated), SW948 (MSS, Dukes' type C, grade III, tumorigenic, p53 mutated), LS1034 (MSS, Dukes C, mutations in p53 (G245S), APC (E1309fs*4) and KRAS (A146T). The human embryonic kidney cell line HEK293 used for E2F1 overexpression was also re-authenticated via STR analysis.

Cells were harvested by scraping the flasks with 1 ml lysis buffer and total RNA was extracted using GenElute Mammalian Total RNA Miniprep Kit (Sigma-Aldrich, St. Louis, MO, cat.no. RTN350) according to the manufacturer's instructions and the RNA integrity was assessed by a Bioanalyzer (RIN> = 9.9). RNA was analyzed on U133plus2.0 or ExonST1.0 arrays (Affymetrix), comparison analysis was performed using MAS5.0 software. Probes accompanied by an Inc/Dec call and a log2 ratio |>0.5| were included, but excluded when listed as ‘‘absent’’. Genes were annotated using the Affymetrix NETAFFX annotation (NCBI Build 36.1, netaffx-build = 28). Exon Array data were quantile-normalized by using the Exon16 algorithm with core transcripts (17881 transcripts) and antigenomic background probes or the iterPLIER expression console. All data analysis was performed using GeneSpring GX 10 software (Agilent).

### Colon Tissue Samples

Total RNA was purified from serial cryosections with more than 75% tumor content using RNeasy MinElute columns following the manufacturer's instructions (Qiagen). Good RNA quality (RIN >7) was verified by analysis on the 2100 Bioanalyzer (Agilent). Analysis on U133A and U133plus2.0 GeneChips and normalization of data was performed as previously described (10). Expression values are given in “log2”. For Exon 1.0 ST Array analyses samples were labeled according to the GeneChip Whole Transcript (WT) Sense Target Labeling Assay Human and hybridized to Exon 1.0 ST Arrays (Affymetrix) as previously described (11). Exon Array data were quantile-normalized by using the ExonRMA16 algorithm with core transcripts (17881 transcripts) and antigenomic background probes. All data analysis was performed using GeneSpring GX 10 software (Agilent).

### cDNA Synthesis

In vitro transcription and labelling of cRNA, hybridisation to Affymetrix U133plus2.0 GeneChips and scanning of these was performed using standard procedures, see reference (12). Comparison analyses of cell line data were performed using Affymetrix MAS5.0 software. Filters applied: Probes were included if the comparison listed an Inc/Dec call accompanied by a log2 ratio >0.5 or <−0.5 (threshold of log>0.5 or log2<−0.5 was arbitrarily defined). Probes were excluded from analysis if they were listed as ‘‘absent’’ and/or had expression values <log2 4 in both samples compared. Genes were annotated using the Affymetrix NETAFFX annotation (NCBI Build 36.1, netaffx-build = 28).

### 5-Aza-2'-deoxycytidine Treatment of Colon Cancer Cells

KRT23 negative HCT116 or DLD1 colon cancer cells were treated with 2.5, 5 or 7.5 µM 5′-AZA-dC in DMSO or CH3COOH (Sigma-Aldrich, Copenhagen, Denmark) every 24 hours for 2 days followed by a reconstitution day as described in the literature.

### Reverse Transcription Quantitative PCR (RTqPCR)

RNA was extracted from HCT116 colon cancer cells treated with different concentrations of AZA using RNeasy columns according to the manufacturer's instructions (Qiagen). cDNA from these samples was synthesized as described (13), using oligo-dT primer instead of random primers. RTqPCR analysis was performed in triplicates on a ABI 7500 Fast Real Time System (Applied Biosystems) using the TaqMan® probe assay Ker23 ID Hs0021096_m1 as recommended by the manufacturer. Data were normalized against Ubiquitin C (UBC) as previously described [19].

### shRNA-vector Preparation, Lentivirus Production and Lentiviral Infection

shRNA-vector preparation, lentivirus production and lentiviral infection was performed as previously described (9). For each of the three cell lines SW480, SW948 and LS1034, a stable control cell line containing an empty lentiviral vector and five different KRT23 specific stable knockdowns were established; the two most efficient sh-1010 and sh-1506 were used for further studies. In brief, the shRNA vector constructs were produced with pLKO.1 puro (kindly provided by Sheila Stewart, Washington University, USA) containing a 1.8 kb stuffer sequence in place of the shRNA cassette. The pLKO.1 plasmid was doubly digested by EcoRI and AgeI for 1 hour to release the 1.8 kb stuffer fragment. DNA fragments were then separated using a 1% agarose gel. The 7.1 kb EcoRI/AgeI band was excised and DNA was extracted using Qiaquick gel extraction kit (Qiagen, Hilden, Germany). Pairs of sense and antisense hairpin oligonucleotides were obtained from Eurofins MWG Operon (Ebersberg, Germany). To form the shRNA cassette 5.4 µg of each oligonucleotide was annealed in a volume of 100 µl. The annealing buffer was 1x T4-DNA ligase buffer (Invitrogen, Karlsruhe, Germany). The annealing mixture was incubated in a thermal cycler (DNA Engine Opticon®2 cycler MJ Research, Waltham, MA, USA), gradually cooling from 99°C to 16°C over 70 min. Ligation was performed in 20 µl reaction volume using 1 unit of T4 DNA ligase (Invitrogen, Karlsruhe, Germany) and 100 ng of vector DNA in a 1∶4 molar vector-insert ratio and incubated at 16°C over night. 2 µl of ligation mixture was used to transform 40 µl competent TOP10 cells (Invitrogen, Karlsruhe, Germany) using standard electroporation procedure. The transformed cells were recovered in 0.8 ml of LB for 1 h at 37°C and plated on ampicillin (100 µg/ml) containing agar plates. Plasmid DNA preparations were made from over-night cultures of individual colonies using the Pure Yield® Plasmid MidiPrep System (Promega, Mannheim, Germany) following the manufacturer’s protocol. The correct sequence was verified via standard cycle sequencing for each shRNA cassette. Lentiviruses were made by transfecting packaging cells (HEK293T) with a 3-plasmid system. DNA for transfections was prepared by mixing 12 µg pCMVΔRR8.2, 1 µg pHIT G and 12 µg pLKO.1 plasmid DNA with 62 µl of 2 M CaCl_2_ in a final volume of 500 µl. Subsequently 500 µl of 2×HBS phosphate buffer was drop wise added to the mixture and incubated for 10 min at RT. The 1 ml transfection mixture was added to 50% confluent HEK293T cell seeded the day before into a 10 cm well plate. Cells were incubated for 16 h (37°C and 10% CO_2_), and the media was changed to remove remaining transfection reagent. Lentiviral supernatants were collected at 36 h post-transfection and for each infection 3 ml supernatant containing 4 µg/ml polybrene was immediately used to infect target cells seeded the day before in 6 cm well plates to reach 70% confluency on the day of infection. Cells were incubated for 24 h, and the media was changed to remove virus particles. To control infection rate a parallel infection under the identical conditions targeting the same cell line was prepared using a lentiviral GFP expression control vector (pRRLU6-CPPT-pSK-GFP, kindly provided by S. Stewart). 6 days after infection 2 µg/ml puromycin was added to the cell culture media. Quantitative RT-PCR was used to validate efficient knockdown and data were normalized against GAPDH, HPRT1 or PPIA. Total RNA from stably transfected cell lines was isolated by acid phenol extraction. cDNA was synthesized using 2 µg of total RNA, oligo(dT)_18_ primers and SuperScript™ II RNase H^−^ reverse transcriptase (Invitrogen, Karlsruhe, Germany) following the manufacturer’s protocol and diluted to a final volume of 50 µl with 1x first strand buffer. Intron spanning primer sets for qRT-PCR were designed using Primer Express 2.0 software (Applied Biosystems, Foster City, CA, USA). qRT-PCR was performed using a SYBR Green I reaction mixture containing 75 mM Tris-HCl (pH 8.8), 20 mM ammonium sulfate, 0.01% (v/v) Tween 20, 2 mM magnesium chloride (all Sigma-Aldrich, Munich, Germany), 1 µl of a 600-fold dilution of SYBR Green I (BioWhittaker, Rockland, ME, USA), 2.5 U Taq polymerase (NEB, Frankfurt a.M., Germany), 0.2 mM dNTP (Promega, Mannheim, Germany) and 0.2 µM of forward and reverse primer (QIAgen, Hilden, Germany) in a final reaction volume of 20 µl. Reactions were run on a DNA Engine Opticon®2 cycler (MJ Research, Waltham, MA, USA). The cycling conditions consisted of 3 min initial denaturation at 94°C and 40 cycles of 94°C for 30 sec, 60°C for 30 sec, 72°C for 30 sec and 80°C for 3 sec. Fluorescence was measured at the last step of each cycle. Melting curves were obtained after each PCR run and showed single PCR products. cDNAs were run in triplicate, non-RT (without reverse transcriptase) and no-template controls were run in duplicates. PCR efficiencies were determined using serial dilutions of a cDNA derived from cell line SW480 Expression levels for genes of interest and for housekeeping genes were measured for in independent PCR runs. Expression ratios were calculated as described by M. Pfaffl [20] using the geometric mean expression of the housekeeping genes GAPD, HPRT1 and PPIA to normalize the expression data for the gene of interest.

### Western Blotting

Western blotting was performed as previously described [21]. The polyclonal rabbit anti-K23 antibody, described in detail in [14], was used in a 1∶500 and 1∶1000 dilution. A monospecific antibody was generated by affinity-purification against the peptide CKWHQQRDPGSKKDYS, position 106–120 in protein sequence NP_056330.3 (Eurogentec, Belgium). The monospecific,anti-K23 antibody was used in a 1∶150 dilution for western blotting. BioRad’s “All Blue” was used as molecular weight marker, beta-actin monoclonal antibody (#A-1978, clone AC-15, Sigma-Aldrich Denmark A/S) diluted to 0.05µg/mL was used as loading control. Mouse monoclonal anti-human MRE11A antibody (ab214, Abcam, UK, lot 912394) was used in 1∶500–1∶1000 dilutions, mouse monoclonal anti-human RAD51 (H-92) (sc-8349, Santa Cruz, USA, lot E0610) in a 1∶100 dilution, mouse monoclonal anti-human BRCA1 (MS110) (ab16780, Abcam, UK, lot GR3646-1) in a 1∶200 dilution. The mouse monoclonal anti-E2F1 was a kind gift from Prof. Kristian Helin, BRIC, Copenhagen, Denmark and was used in a 1∶5 dilution. Extracts from HEK293-cells overexpressing recombinant HA-tagged E2F1 from the expression vector pCMVHA-E2F1 were used as a control [22].

### Immunofluorescence Microscopy

Cells were seeded on 16 well chamber slides (Nunc, glass cat. No. 178599; no endogenous fluorescence), fixed in cold methanol (-20 C) and stained with the following antibodies: rabbit polyclonal anti-K23 antibody (1∶500) (11), mouse monoclonal anti-KI67 (1∶100, DAKO Cytomation), anti-E2F1 undiluted, anti-MRE11A 1∶1000, anti-RAD51 1:50 and anti-BRCA1 1:200. The secondary antibodies used were AlexaFlour 488 goat anti-rabbit IgG highly cross-adsorbed (1∶2,000; Mol. Probes Inc., Eugene, OR) or AlexaFlour 488 goat anti-mouse IgG highly cross-adsorbed (1∶2,000; Mol. Probes Inc., Eugene, OR). Hoechst 33342 was used for nuclear stains and slides were mounted with Fluorescence Mounting Medium (DakoCytomation) and a coverglass (Nunc, Cat No. 171080). Images were acquired with an Axiovert 200 M (Zeiss Microimaging, Inc.).

### Ingenuity Pathway Analysis (IPA)

Microarray expression data normalized with RMA (Robust Multichip Average) were subjected to IPA, version 8.8. Data below the arbitrarily set threshold of log2<4.0 were excluded from analyses, log2 intensities of log2<5 were regarded as absent expression. Expression values were normalized around zero. Normalized ratios given as (−INF, −1] and [1, +INF) were submitted to IPA.

### Proliferation Studies

Viability and proliferation of colon cell lines stably transfected with sh-RNA against KRT23 was assessed by an MTT assay (3-[4,5-dimethylthiazol-2-yl]2,5-diphenyltetrazolium bromide) as a function of cellular metabolism according to the manufacturer’s instructions (Roche, Germany). Absorbance at 550 nm/690 nm was measured at different points of time. Proliferation of colon cancer cells was assessed by the CyQUANT® NF assay according to the manufacturer’s instructions (Invitrogen). Fluorescence intensities were measured with a Synergy™ HT Microplate Reader (Biotek, Germany) using excitation at 485/20 nm and fluorescence detection at 528/20 nm. Cell cytotoxicity was assessed by a LDH-assay (lactate dehydrogenase) according to the manufacturer’s instructions (Cytotoxicity Detection kit, Cat. No. 11644 793001, Roche Diagnostics, Hvidovre, Denmark). Label-free monitoring of proliferation and viability over a range of several days was performed on 96-well E-plates on an RTCA (Real Time monitoring of cells) SP Single Plate instrument or 16-well E-plates or CIM plates (cellular invasion and migration) on a DP Dual Plate instrument (Roche). Adhesion was monitored using E-plates in intervals of 1–5 minutes within the first 1–6 hours after seeding. Proliferation was monitored using E-plates in intervals of 15 min within periods of 1–120 hours, seeding 4000–16000 cells per well, respectively. Analyses were performed in triplicates and results were validated by conventional assays. Cell migration on CIM-plates was monitored in duplicates in 1 minute intervals within periods of 2–48 h hours, seeding 40.000–60.000 cells per well. Using the intrinsic RTCA software, the doubling time (DT) was calculated according to DT = log2/slope published by Zhang et al [23], http://www.bioconductor.org/packages/release/bioc/html/RTCA.html. The calculated DT is the Cell Index Doubling Time, which is not equal to the time when the cell number doubles, because Cell Index values are a combination of the measure of cell viability, cell number, cell morphology, and the degree of cell adhesion.

### Irradiation of Colon Cancer Cells

To ascertain whether irradiation may have an impact on SW948 or LS1034 cells with a KRT23 knockdown we performed a dosage optimization irradiating with 0–10 GY of γ-rays using a ^137^Cs radiator Gammacelle 2000RH (AEK Risø, Denmark) as previously described [24].

### Statistical Analysis

Statistical analysis was performed using STATA 10 (Statacorp, Texas, USA). Transcript values were expressed as median log2± standard deviation (sd). A two-tailed Student’s t-test was applied and p-values p<0.05 were considered as statistically significant.

## Results

### KRT23 Promoter Methylation

**Figure 1:**
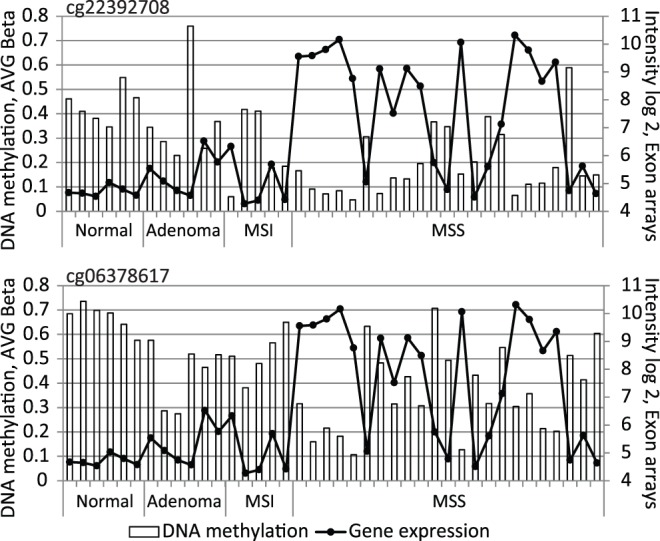
Methylation versus expression profiling of KRT23. Comparison of KRT23 transcription data from Exon 1.0 ST arrays (–•–) to methylation data from 2 probes, cg22392708 and cg06378617 from the Illumina Bead arrays (□) showed a negative correlation between methylation and transcription in the 40 tissue samples analyzed (Spearman rank correlation coefficient of −0.64 and −0.74, respectively).

**Figure 2:**
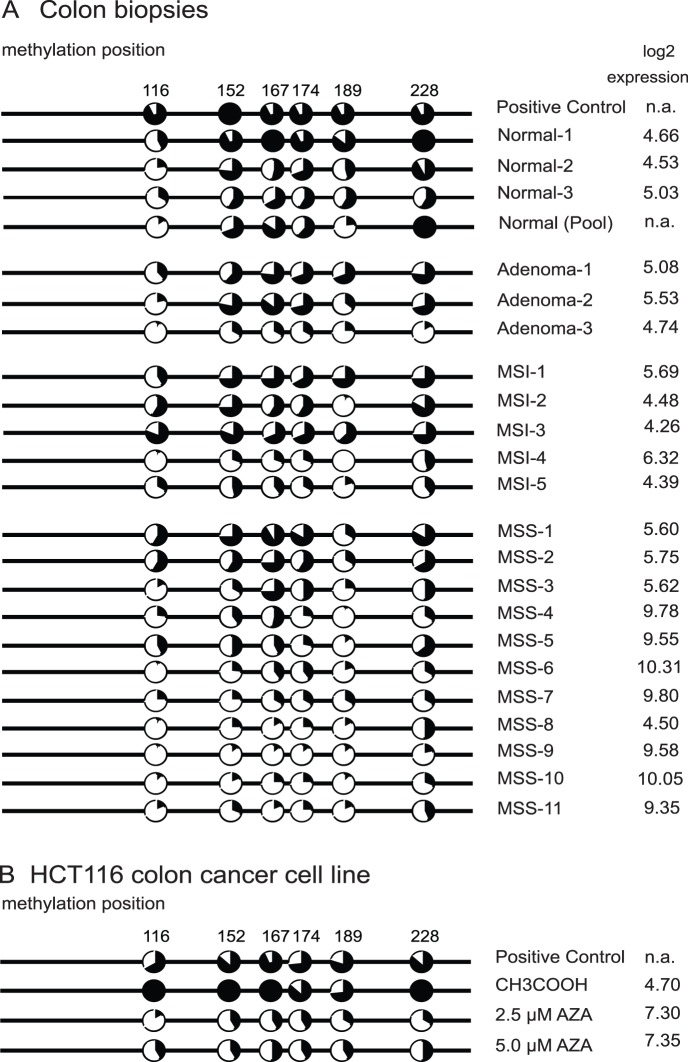
Bisulfite sequencing of colon tissues and HCT116 cells six positions. Position 116 corresponds to Cg22392708. • methylated, ○ unmethylated; MSI and MSS colon adenocarcinomas. A) Methylation status of colon biopsies was compared to transcript expression data from Exon 1.0 ST arrays. For the majority of samples, high methylation values are in agreement with low expression values and vice versa. Log2 intensities are shown in the panel on the right, values below log2 = 5 were regarded as absent expression. B) Treatment of HCT116 cells (MSI) not expressing KRT23 with 5′-AZA-dC showed that decreased methylation resulted in an increased expression of the KRT23 transcript.

The methylation status of the KRT23 promoter was assessed in 40 colon tissue samples (six normal mucosa, six adenoma, five MSI and 23 MSS adenocarcinoma tissues) using Illumina Bead arrays interrogating CG-sites at position cg06378617 and cg22392708 located in the KRT23 promoter (Figure A in Figure S1 in File S1). A highly significant decrease in the methylation levels of MSS adenocarcinomas could be observed for both interrogated CG-sites when compared to six normal mucosa samples (Mann-Whitney U-test, p-value 1.9E-04 for cg06378617 and 5.3E-04 for cg22392708). MSI adenocarcinomas as well as adenomas showed significant reduced methylation for the cg06378617 CG site only (Mann-Whitney U-test, p-value 1.7E-02 and 2.2E-03, respectively). Parallel transcript expression profiling of the same samples using Exon 1.0 ST arrays showed that the KRT23 transcript was absent in normal mucosa, confirming previous results (8). However, unambiguous transcript levels were identified in 4/6 adenomas and in the majority of adenocarcinomas. A negative correlation was identified between promoter methylation and KRT23 transcript expression at positions both interrogated CG sites, position cg06378617 and cg22392708 with Spearman rank correlation coefficient of −0.64 and −0.74, respectively (Figure 1). Illumina array data were validated by bisulfite sequencing using samples with different KRT23 expression levels ranging from low to high from an independent sample set not previously analyzed on Illumina arrays, and where DNA samples and expression microarray data were available. As it was not possible to obtain specific primers for the Illumina CpG-site cg06378617, we analyzed promoter methylation at cg22392708 (position 116 in Figure 2A) and at five additional adjacent CpG-sites (positions 152, 167, 174, 189 and 228). Bisulfite sequencing of at least 10 clones per sample was performed on DNA extracted from 23 tissue samples comprising normal mucosa (n = 3), a normal pool (n = 10), adenomas (n = 3) and adenocarcinomas (MSI n = 5 and MSS n = 11) (Figure 2A). Then, methylation status was compared to microarray transcript profiling data. In all positions, except at position 116, high level of methylation was accompanied by low KRT23 expression in 87% of the cases. The KRT23 promoter of normal mucosa showed >50–75% methylation accompanied by absent KRT23 expression (transcript levels of log2<5). In contrast, the majority of the MSS tumors showed less than 25% methylation accompanied by high KRT23 expression levels of log2>9 in 7/11 MSS tumors analyzed (Figure 2A).

### KRT23 Expression is Induced by Demethylation

Two MSI colon cell lines, HCT116 and DLD1 without KRT23 expression, were treated with increasing concentrations of 5-aza-2′-deoxycytidine (5′-AZA-dC) and DMSO or CH3COOH as controls and cell viability was monitored by MTT assay (not shown). RTqPCR analysis either using a SYBR-green probe or a Taqman probe against KRT23 showed that 2.5µM 5′-AZA-dC was sufficient to induce a strong upregulation of KRT23 resulting in an 18-fold (HCT116 cells) or 120-fold (DLD1 cells) increase, respectively, compared to mock treated cells (Figure B in Figure S1 in File S1). Whole genome expression profiling using Exon 1.0 ST arrays confirmed the strong upregulation of KRT23 in HCT116 and DLD1 cells upon 5′AZA-dC treatment and showed the re-expression of several genes as e.g. MAEL and UCHL1 (data not shown), genes previously reported to be inducible with a demethylating agent [25]. Bisulfite sequencing at six positions in the KRT23 promoter of the 5′-AZA-dC treated HCT116 cells confirmed an 80%–100% methylation in the CH3COOH treated controls, while methylation was decreased to 25%–50% methylation upon treatment with 2.5–5.0 µM 5′-AZA-dC (Figure 2B). In conclusion, the KRT23 transcript expression is 5′-AZA-dC inducible suggesting a potential epigenetic regulation for KRT23.

### Lentiviral Mediated Stable Knockdown of KRT23 in Colon Cancer Cell Lines

As early stage adenocarcinomas already express moderate to high levels of KRT23 *in vivo* [14], we wanted to know whether depletion of KRT23 may affect the molecular and cellular functions of colon cancer cells. In an initial approach, lentiviral mediated knockdown of KRT23 was applied to the human colon cancer cell line SW480. Five different shRNA sequences targeting KRT23 were analyzed, where the most efficient constructs were sh-1010 and sh-1506 (Figure A in Figure S2 in File S1). Transcript profiling using U133 2.0plus arrays was performed on extracts from SW480 cells stably transfected with sh-1506 or sh-1010, and compared to control cells stably transfected with an empty vector. Construct sh-1010 resulted in 3968 genes differentially expressed (log2>|0.5|) upon KRT23 depletion, while construct sh-1506 with a knockdown efficiency of about 90% resulted in 7156 genes altered, hereof 3145 (log2>|0.5|) target genes in common for both knockdown constructs, increased or decreased in both analyses in the same direction. Construct sh-1506 was further used to study the effect of KRT23 knockdown in three different colon cancer cell lines.

### Expression Profiling of KRT23 Depleted Cell Lines

**Figure 3:**
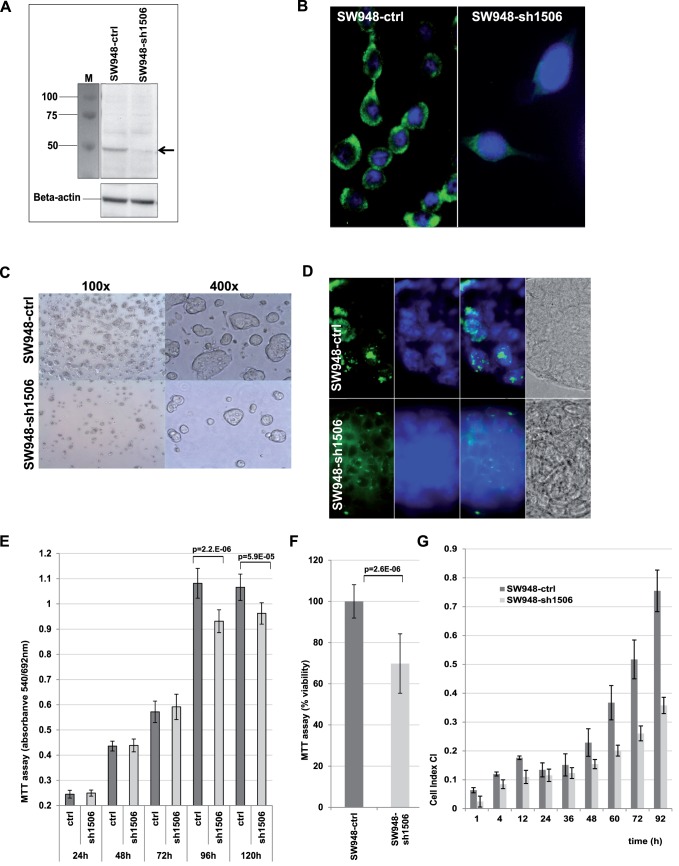
Knockdown of KRT23. **A**) Western Blot of freshly made SW948-ctrl and SW948-sh1506 cells extracts with 20 µg extract per lane using a monospecific anti-K23 antibody in a 1∶150 dilution, Marker Biorad All Blue. Stable knockdown of K23 in the SW948-sh1506 cells resulted in >80% reduced K23 protein expression compared to the SW948-ctrl cells. Beta-actin was used as loading control. B) Immunofluorescence analysis confirmed a decreased K23 expression in SW948-sh1506 cells; anti-K23 antibody 1∶500, detection with Alexa 488, nuclear stain Hoechst, magnification 630x C) Visual inspection indicated a lower cell density for SW948-sh1506 cells 48 h post seeding. D) SW948-sh1506 cells showed less nuclear expression of the proliferation marker KI67 (green); anti-KI67 1:100 E) SW948-ctrl and SW948-sh1506 cells were seeded on 96-well plates with 4000 cells per well (n = 12) and proliferation was analyzed post-seeding at five time-points. Proliferation of SW948-sh1506 cells was significantly (Fisher’s exact t-test, p<0.0001) decreased at 96 hours and 120 hours post-seeding. F) The MTT assay was repeated by seeding 16000 cells per well (n = 11) on a 96 well plate and proliferation/viability was analyzed at 48 h post-seeding and percentage viability was measured. The proliferation of KRT23 depleted cells was significantly (p = 2.6E-06) decreased by about 30%. G) SW948-ctrl and SW948-sh1506 cells were seeded on 96-well RTCA-plates with 8000 cells/well (n = 3). Values are shown as medians and standard deviations for each group at selected time points for a representative experiment.

In an extended approach we used three different MSS colon cell lines with low to moderate (SW480 cells) or high KRT23 expression (SW948 and LS1034 cells). Each cell line was stably transfected with the sh-1506 construct, and KRT23 expression was compared to the corresponding control cells with an empty vector, knockdown efficiencies were assessed by RTqPCR (Figure B–D in Figure S2 in File S1). Whole genome transcript profiling was performed on Affymetrix Exon 1.0 ST arrays and the RMA-normalized KRT23 expression data are shown in Figure E in Figure S2 in File S1. KRT23 knockdown in SW948 cells decreased the KRT23 level from log2 = 9.15 to log2 = 6.97 (log2 ratio -2.18), and to a lesser extent in LS1034 cells (log2 ratio -1.29) and SW480 cells (log2 ratio -1.15). Western blotting of SW948 cell extracts using the previously characterized polyclonal anti-K23 antibody [14] showed that the knockdown decreased the K23 protein expression, thereby affecting different molecular isoforms of K23 ranging from less than 20 kDa to more than 90 kDa (Figure 3A). The previously identified about 47 kDa protein was strongly expressed in SW948 cells and knockdown decreased the protein expression by about 50%, while the additional isoforms were decreased by about 80%. Immunofluorescence analysis (Figure 3Aa) supported these findings of a decreased K23 expression in SW948-sh1506 cells compared to the control; still some protein expression was detectable (Figure 3B). KRT23 knockdown lead to differential expression of 3647 (SW948) or 4491 transcripts (LS1034), respectively applying a threshold of log2>|0.5| to the RMA normalized data (Table 1). A comparison of the genes differentially expressed identified 970 genes in common in two cell lines, SW948-sh1506 and LS1034-sh1506, showing increased or decreased expression of a transcript in the same direction with a threshold of log2>|0.5|. There was less accordance to SW480 cells and further analyses were performed on SW948 and LS1034 cell lines only.

**Table 1:** Colon cancer cells depleted from KRT23 compared to control cells.

RMA normalized data were subjected to IPA analysis						
Molecules differentally expressed upon KRT23 knockdown						
	log2>|0.5|		log2>|1.0|		log2>|2.0|	
	up	down	up	down	up	down
**SW948**	1060	2587	206	767	32	192
**LS1034**	3166	1325	837	240	70	13
**SW480**	2072	1619	542	156	5	4
**Molecular and Cellular functions log2>1.0 or <**−**1**						
	**SW948**		**LS1034**			
	**p-value**	**molecules**	**p-value**	**molecules**		
Cell Cycle	1.79E-45	149	1.92E-07	79		
DNA Replication, Recombination, and Repair	1.73E-14	139	5.41E-08	65		
Cellular Assembly and Organization	1.66E-12	69	1.97E-05	15		
Cell Death	4.67E-10	151	4.49E-05	130		
Cellular Growth and Proliferation	1.67E-08	160	2.97E-04	146		
**Top Canonical Pathways log2>1.0 or <−1**						
	**SW948**		**LS1034**			
	**p-value**	**ratio** *	**p-value**	**ratio** *		
Role of BRCA1 in DNA Damage Response	2.58E-20	26/58	4.12E-04	10/58		
Cell Cycle Control of Chromosomal Replication	2.25E-12	14/30	3.94E-06	9/30		
ATM Signaling	8.85E-11	17/54	<0.05	5/54		
Role of CHK Proteins in Cell CycleCheckpoint Control	7.08E-11	14/53	<0.05	4/53		

* number of molecules differentially expressed to number of molecules in given pathway.

### KRT23 Knockdown Affected Molecular and Cellular Functions

The RMA normalized expression data were subjected to Pathway analysis. KRT23 depletion mainly affected molecular and cellular functions within the Cell Cycle, DNA Replication, Recombination and Repair. Moreover, canonical pathways as “Role of BRCA1 in DNA Damage Response”, “Cell Cycle Control of Chromosomal Replication”, “ATM signaling” and “Role of CHK Proteins in Cell Cycle Checkpoint Control” were identified (Table 1). The top 50 genes involved in Cell cycle regulation (Table S1 in File S1) or cellular proliferation (Table S2 in File S1) differentially expressed in SW948-sh1506 and LS1034-sh1506 cells compared to their control are listed. The proliferation marker MKI67 was strongly decreased suggesting that KRT23 depletion resulted in a decreased proliferation. In vitro functional analyses confirmed this, showing a reduction in the total number of cells upon KRT23 knockdown (Figure 3C). Further, immunofluorescence microscopy showed that the typical granular nuclear staining of the proliferation marker KI67 was decreased in SW948-sh1506 cells (Figure 3D), correlating with the KI67 transcript data (Table S2 in File S1). MTT assays showed that KRT23 knockdown significantly (t-test, p<0.05) decreased the cell viability of SW948-sh1506 cells at 96 hours post-seeding (Figure 3E). Analysis of these cells by an LDH assay showed that the decreased cell viability was not caused by any kind of cell death (data not shown). Thus the proliferation of SW948-sh1506 cells decreased by about 30% compared to SW948-ctrl cells (Figure 3F). Moreover, X-Celligence RealTime Cell monitoring (RTCA) showed that the proliferation of SW948-sh1506 cells was decreased compared to SW948-ctrl cells (Figure 3G). When 8000 cells were seeded, the calculated Cell Index Doubling Time was 24.28 hours (SD 0.17) for the SW948-ctrl cells and 37.16 hours (SD 0.16) for the SW948-sh1506 cells with KRT23 knockdown [76h interval, determined 24 h-100 h post-seeding]. When 4000 cells were seeded the calculated Cell Index Doubling Time was 18.98 hours (SD 0.13, SW948-ctrl) and 25.25 hours (SD 0.04, SW948-sh1506 cells) [76 h interval, 43 h-119 h post-seeding]. In conclusion, in SW948-sh1506 cells proliferation was decreased by about 25% to 35% compared to SW948-ctrl cells with an empty vector being in accordance with the results obtained by MTT assays. Furthermore, these results were strongly supported by similar findings obtained with two additional colon cancer cell lines, namely LS1034 and SW480 cells. Both cell lines showed a significantly (p<0.05) decreased proliferation upon KRT23 knockdown as shown by RTCA analysis as well as MTT assays at different time points, details are shown in Figure S3 in File S1.

### KRT23 Depletion Impacts Cellular Repair Systems

**Figure 4:**
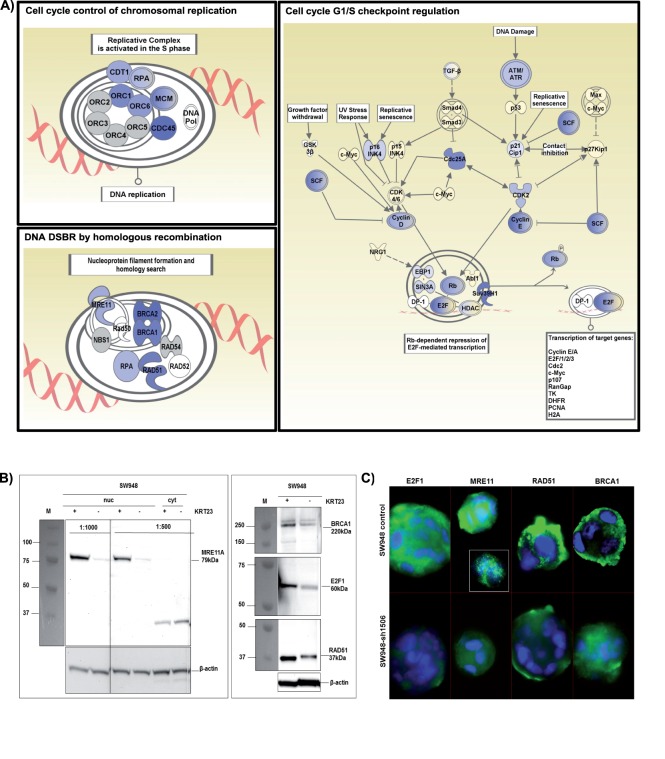
Ingenuity pathway analyses and protein expression. A) KRT23 knockdown affected main regulatory pathways, and decreased the expression of key molecules. Key molecules decreased upon KRT23 knockdown essential for the assembly of replication and repair complexes comprise E2F1, BRCA1, BRCA2, RAD51, MRE11A and RPA. blue = decreased, yellow = increased transcript expression. B) Western blotting. MRE11A, E2F1, BRCA1 and RAD51 proteins were markedly decreased in KRT23 depleted SW948-sh1506 cells compared to control cells. 20µg nuclear (nuc) or cytoplasmic extract (cyt) was loaded. Anti-MRE11A antibody 1∶1000 dilution (nuclear extracts), 1∶500 dilution (nuclear and cytoplasmic extracts). Marker All Blue BioRad; C) Immunofluorescence staining of SW948-ctrl and SW948-sh1506 cells also showed that KRT23 knockdown resulted in a decreased expression of E2F1, MRE11A, RAD51 and BRCA1.

Pathway analysis applied on genes consistently affected in both SW948 and LS1034 cells showed that KRT23 knockdown mainly affected molecules involved in DNA repair and chromosomal replication. Three different pathways, namely “Cell cycle control of chromosomal replication”, “cell cycle G1/S checkpoint regulation” and “DNA double strand break repair (DSBR) by homologous recombination” are depicted in Figure 4A. Selected molecules involved in DSBR and mismatch repair differentially expressed in both cell lines, SW948-sh1506 and LS1034-sh1506 compared to their control cells are shown in Table 2, an extended list including molecules of the complex initiating the DSBR is shown in Table S3 in File S1. The decrease of four selected key proteins MRE11A, E2F1, RAD51 and BRCA1 upon KRT23 knockdown was confirmed by Western blotting using nuclear extracts from SW948-sh1506 and SW948-ctrl cells (Figure 4B). Moreover, decreased protein expression upon KRT23 knockdown was also confirmed by Immunofluorescence microscopy (Figure 4C).

**Table 2:** KRT23 knockdown affects canonical pathways involved in DNA damage control.

			log2		
Entrez Gene ID	Symbol	Entrez Gene Name	Ratio	SW948-ctrl	SW948-sh1506
	**DSBR - Double strand break repair**				
672	**BRCA1**	breast cancer 1, early onset	−2.120	7.850	5.730
675	**BRCA2**	breast cancer 2, early onset	−2.790	8.050	5.260
3978	**LIG1**	ligase I, DNA, ATP-dependent	−1.480	8.400	6.920
4361	**MRE11A**	MRE11 meiotic recombination 11 homolog A	−1.250	6.500	5.250
5888	**RAD51**	RAD51 homolog (RecA homolog, E. coli)	−2.090	9.090	7.000
6117	**RPA1**	replication protein A1, 70 kDa	−1.100	9.380	8.280
	**Mismatch Repair**				
9156	**EXO1**	exonuclease 1	−3.23	8.99	5.76
4436	**MSH2**	mutS homolog 2, colon cancer, nonpolyposis type 1	−1.89	8.86	6.97
5111	**PCNA**	proliferating cell nuclear antigen	−1.18	11.53	10.35
5424	**POLD1**	polymerase (DNA directed), delta 1	−1.04	8.05	7.01
5982	**RFC2**	replication factor C (activator 1) 2, 40 kDa	−1.34	10.00	8.66
5983	**RFC3**	replication factor C (activator 1) 3, 38 kDa	−1.89	10.55	8.66
5985	**RFC5**	replication factor C (activator 1) 5, 36.5 kDa	−2.34	9.16	6.82
6117	**RPA1**	replication protein A1, 70 kDa	−1.10	9.38	8.28

Data were obtained by microarray expression profiling followed by RMA normalization, comparison of SW948 control cells versus SW948-sh1506 with KRT23 knockdown. All molecules are located in the nucleus.

### Response to Serum Starvation

To exclude that the effects seen upon KRT23 knockdown were mainly a consequence of growth inhibition, and to ensure that the identified molecules were mainly targets of KRT23 knockdown, LS1034-ctrl and SW948-ctrl cells as well as their corresponding sh1506-KRT23 knockdown cell lines were subjected to serum withdrawal conditions (10% serum versus serum free). The four cell lines showed a significant response to serum withdrawal and clearly slowed down proliferation at 24 h or 48 h, respectively, as monitored by MTT-assays (Figure S4 in File S1). SW948-ctrl cells were incubated under 10% serum or serum-free conditions, harvested at 24 h post-seeding and subjected to expression profiling using Exon 1.0 ST arrays (n = 4). RMA-normalized data were submitted to IPA. Serum starvation resulted in 294 genes differentially expressed (log2 ratio >|0.5|, hereof 39 with a log2 ratio >|1.0|), mainly affecting Cellular Growth and Proliferation (1,8E-07, n = 47), Cellular Movement (3,2E-07, n = 25) and Cell Death (2E-05, n = 32). Genes differentially expressed upon starvation were compared to genes involved in DNA replication and repair being affected upon KRT23 knockdown (Table S3 in File S1). However, Tenascin-C (TN-C) was the only gene strongly affected in both approaches. It is known that TN-C expression levels correlate with cell cycle progression [26] and is not regarded as a target of KRT23 knockdown. In conclusion, neither the “mismatch repair pathway” nor the “double strand break repair homologous recombination pathway” was affected upon serum withdrawal, and therefore the effects on DNA replication and repair seem to be caused by KRT23 knockdown per se.

### Ionizing Radiation of Colon Cancer Cells

**Figure 5:**
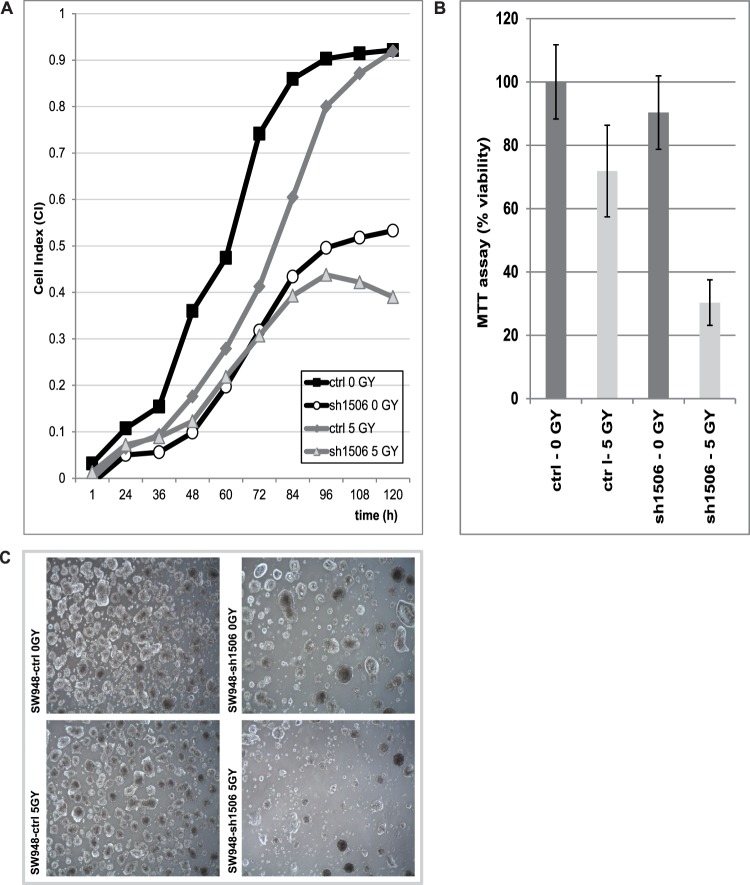
Irradiation of colon cancer cells. A) SW948-ctrl or SW948-sh1506 with stable KRT23 knockdown were irradiated with 0 GY or 5 GY of γ-rays, respectively and seeded on RTCA16-well plates with 16.000 cells/well (n = 4). Non-irradiated SW948-sh1506 cells showed a reduced proliferation rate compared to non-irradiated SW948-ctrl cells. Irradiated SW948-ctrl cells continued proliferation after a short lag period, while the proliferation of the irradiated KRT23-depleted SW948-sh1506 cells decreased after 72 h post-irradiation. The Cell Index CI of the irradiated SW948-sh1506 cells markedly dropped down at about 96 h post-irradiation suggesting a detaching of the cells, possibly induced by cell death upon irradiation of the KRT23 depleted cells. B) A MTT viability assay co-performed at 120 h post-irradiation together with RTCA showed that the viability of KRT23 depleted SW948-sh1506 cells was reduced by 60% upon irradiation with 5GY (p = 8.1E-08) compared to 30% in the SW948-ctrl cells (p = 6.4E-05). C) Visual inspection at 7 days post-irradiation showed a markedly reduced number of cells in KRT23 depleted SW948-sh1506 cells irradiated with 5GY compared to non-irradiated cells.

We hypothesized that a decreased expression of genes encoding proteins involved in DNA repair would increase the irradiation sensitivity, leading to cells being less proficient in repair of double strand breaks upon irradiation. SW948 and LS1034 colon cancer cells, either with an empty vector or with a stable KRT23 knockdown, were irradiated with 0 GY or 5 GY of γ-rays. The culture medium was immediately changed after irradiation and cells were seeded for proliferation studies. RTCA analysis (0–146 h post-irradiation) upon irradiation showed that proliferation of control cells continued after a short lag period, while overall proliferation was not affected by irradiation. Interestingly, proliferation of irradiated KRT23 depleted cells was reduced compared to non-irradiated KRT23 depleted cells in SW948 cells, whereas the LS1304 cells, that proliferated much faster, did not show any effect. The Cell Index CI of the irradiated SW948-sh1506 cells decreased at 96 h post-irradiation, indicating induction of cell death upon irradiation of cells with KRT23 knockdown (Figure 5A). MTT-assays measuring cell viability (96–120 h post-irradiation) showed that the viability of KRT23 depleted cells was markedly decreased upon 5 GY irradiation compared to non-irradiated cells. Viability of SW948-ctrl cells decreased to 70% upon irradiation, compared to only 30% of the irradiated SW948-sh1506 cells with KRT23 knockdown (Figure 5B). Light microscopy at 0–18 days post irradiation showed less SW948-sh1506 cells in the irradiated culture dish compared to the non-irradiated culture dishes, confirming the results obtained by RTCA and MTT assays. The effect was still visible at 7 days post-irradiation as shown in (Figure 5C). Furthermore, we also observed a decreased proliferation of the KRT23-depleted LS1034-sh1506 cells upon irradiation using RTCA analysis and MTT assays, the effect was strongest within the first days post-irradiation (data not shown).

## Discussion

This study showed that the KRT23 promoter is partially methylated in normal mucosa, and less methylated in the majority of the MSS tumors. Decreased methylation correlated with increased KRT23 transcript expression. Treatment of colon cell lines with a demethylating agent induced strong KRT23 transcript expression *in vitro.* Expression profiling of shRNA mediated stable knockdown of KRT23 in three different colon cell lines, SW948, LS1034 and SW480 with different KRT23 levels, showed that KRT23 depletion affected molecules of the cell cycle and DNA replication, recombination and repair. *In vitro* analyses confirmed that KRT23 depletion significantly decreased the cellular proliferation of colon cancer cell lines, and markedly decreased the expression of genes involved in DNA damage response, mainly molecules of the double strand break repair homologous recombination pathway. Decreased expression upon KRT23 knockdown was confirmed at the protein level for key molecules MRE11A, E2F1, RAD51 and BRCA1 and knockdown of KRT23 rendered colon cancer cells more sensitive to irradiation.

In a previous study we showed that phosphokeratin KRT23 was strongly upregulated in colon adenocarcinomas compared to normal colon mucosa [14]. In a genome wide methylation profiling study on colon biopsies, we found KRT23 to be among the top 60 dysmethylated candidates of the 21.752 genes analyzed [17]. In this study we provide evidence that the KRT23 promoter is methylated in normal mucosa with no or very low expression of KRT23, while most adenomas and adenocarcinomas with high KRT23 expression were found to be hypomethylated. KRT23 expression was inducible by treatment with a demethylating agent. In conclusion, these results provide evidence for an epigenetic regulation of KRT23 in colon mucosa. However, methylation and expression status did not match for all cases, probably suggesting the existence of an alternative regulatory mechanism. It is noteworthy that the Illumina Bead array CpG-site Cg22392708 (corresponding to position 116) was >60% unmethylated in several samples. The methylation status of this specific position did not correlate to KRT23 expression. Bisulfite sequencing of single clones revealed a very heterogeneous methylation pattern for some of the clones, indicating that some sites are more relevant than others.

Expression profiling was performed on three MSS colon cell lines with different KRT23 expression levels using shRNA mediated stable knockdown of KRT23 followed by RMA normalization. The effect of KRT23 knockdown was strongest in SW948 cells with highest KRT23 expression. Several identical target genes and pathways were identified in at least two out of three cell lines. However, knockdown of KRT23 in SW480 cells was partially deviating from the two other cell lines, e.g. genes downregulated in SW948 and LS1034 were not found to be differentially expressed in SW480 upon KRT23 knockdown and vice versa. A possible explanation may be the relatively low endogenous KRT23 expression together with a different genetic background of the cells. However, functional analyses showed that KRT23 knockdown significantly decreased proliferation in all three cell lines.

KRT23 depletion affected molecules within cell cycle and DNA replication, recombination and DNA damage response. Differential expression of DNA damage response genes may also be caused indirectly by perturbance of cell cycle genes. However, serum withdrawal did not lead to significant changes in genes of the “mismatch repair pathway” or the “double strand break repair homologous recombination pathway”.

At the molecular level, KRT23 knockdown decreased the expression level of several genes involved in the cell cycle G1/S checkpoint such as e.g. E2F1, ATM/ATR, cyclin D and cyclin E. Furthermore, it mainly affected DNA replication and repair, e.g. strongly decreasing the expression of BRCA1, BRCA2, MRE11A, RPA or RAD51. The transcription factor E2F1, previously characterized by the Helin group [27], is involved in cell cycle control and action of tumor suppressor proteins. It interacts with tumor suppressor RB1 and p53 [28], induces cell proliferation upon activation, and can also mediate p53-dependent/independent apoptosis [29]. In conclusion, KRT23 depleted colon cancer cells may be restricted in their assembly of functional G1/S complexes. As a consequence, this may result in decreased transcription of cell cycle proteins for G1/S transition thus markedly slowing down proliferation of the KRT23 depleted cells.

In addition to its cell cycle involvement, E2F1 deficiency also impairs RPA and RAD51 foci formation [30]. RPA and RAD51 are together with BRCA1, BRCA2 and MRE11A (meiotic recombination 11) part of the protein complex initiating DSBR by homologous recombination for repair of severe forms of DNA damage, e.g. damages caused by irradiation. BRCA1 and BRCA2 both control RAD51, which catalyzes homologous pairing and DNA strand exchange [31]. RAD51 is a known target of E2F1, as it has a binding site for E2F1 [32]. MRE11A is part of the initiating MRN-complex (MRE11-RAD50-NBS1 Nijmegen breakage syndrome 1), recognizing the DNA damage and tethering the DNA ends together again [33]. MRE11A is a nuclear protein with exonuclease activity being involved in homologous recombination, telomere length maintenance, and DNA DSBR. Xu et al showed that reduced Mre11 protein levels lead to radiosensitization of human tumor cells [34]. Furthermore Chen et al reported that the absence of E2F1 leads to spontaneous DNA breaks and impaired recovery following exposure to ionizing radiation [30]. These data strongly support our findings, that KRT23 depletion rendered the cells more sensitive to irradiation. In conclusion knockdown of KRT23 in colon cancer cells strongly decreased the expression of several molecules necessary for the establishment of DNA repair complexes, which may result in a less efficient DNA repair upon irradiation damages. As a consequence this will impact the genome maintenance, finally resulting in the death of repair-deficient cells.

## Conclusions

In conclusion, here we show evidence that KRT23 expression is epigenetically regulated in colon mucosa and that KRT23 is upregulated in colon cancer due to demethylation of the KRT23 promoter. KRT23 depletion affected a number of key molecules involved in cell cycle control and DNA damage control. Moreover, the absence of KRT23 decreased cell proliferation and rendered cells more sensitive to irradiation. Taken together, the findings presented here together with the fact that K23 is an intermediate filament protein and is interacting with other proteins may suggest a multifunctional role for K23. However, the molecular function of the K23 protein is still far from being understood.

## Supporting Information

File S1 A combined supporting information file containing supplementary data, three supplementary Tables S1–S3, supplementary figure legends and four supplementary Figures S1–S4.
(PDF)
