# Supplementary material for: Keratin23 (KRT23) Knockdown Decreases Proliferation and Affects the DNA Damage Response of Colon Cancer Cells
Source: PLoS One. 2013 Sep 9;8(9):e73593. doi: 10.1371/journal.pone.0073593 (PMC3767798; doi:10.1371/journal.pone.0073593)

SUPPLEMENTARY File S1

Keratin23 (KRT23) knockdown decreases proliferation and affects the DNA damage response of colon cancer cells

Karin Birkenkamp-Demtröder, Stephan Hahn, Francisco Mansilla, Kasper Thorsen, Abdelouahid Maghnouj, Rikke Christensen, Bodil Øster and Torben Falck Ørntoft

SUPPLEMENTARY DATA

KRT23 associated transcription factor FOXQ1 is induced by 5'-AZA-dC**.** In a previous study, we identified the transcription factor FOXQ1 significantly correlated to KRT23 expression in human colon tissue samples (rs=0.54, 95%CI 0.42-0.65, p=<0.0001) and microarray data of >160 colon samples showed that FOXQ1 was strongly upregulated in MSS colon adenocarcinomas (log2 10.17) compared to normal mucosa (log2 2.6) [1]. Furthermore, knockdown of FOXQ1 resulted in a decrease in p21(CIP1/WAF1) and upregulation increased tumorigenicity [2]. In this study microarray expression profiling of 5'-AZA-dC treated colon cell lines showed that FOXQ1 was induced by 2.5 µM 5'-AZA-dC in HCT116 but not in DLD1 cells.

SUPPLEMENTARY REFERENCES

1. Birkenkamp-Demtroder K, Mansilla F, Sørensen FB, Kruhøffer M, Christensen L, Aaltonen LA, Verspaget H., Ørntoft TF (2007) Phosphoprotein Keratin 23 accumulates in MSS but not MSI colon cancers in vivo and impacts viability and proliferation in vitro. Molecular Oncology Vol 1, Issue 2: 181-195.

2. Kaneda H, Arao T, Tanaka K, Tamura D, Aomatsu K, Kudo K, Sakai K, De Velasco MA, Matsumoto K, Fujita Y, Yamada Y, Tsurutani J, Okamoto I, Nakagawa K, Nishio K (2010) FOXQ1 is overexpressed in colorectal cancer and enhances tumorigenicity and tumor growth. Cancer Res 70: 2053-2063.

SUPPLEMENTARY TABLES

Table S1 50 genes affecting the cell cycle upon KRT23 knockdown in SW948 cells. Data were compared to expression in LS1034-ctrl and LS1034-sh1506 cells. Data were obtained by microarray expression profiling followed by RMA normalization, comparison of SW948 control cells versus SW948-sh1506 with KRT23 knockdown (treshold log2 ratio >|2.0|). Some genes affected in SW948 were not affected or to a lesser extent affected in LS1034 cells.

			log2						
**Entrez Gene**	**Symbol**	**description**	**ratio**	**SW948-ctrl**	**SW948-sh1506**	**ratio**	**LS1034-ctrl**	**LS1034-sh1506**	**Location**
54821	**ERCC6L**	excision repair cross-complementing rodent repair deficiency, (	-3.6	7.9	4.2	-0.8	5.5	4.7	Nucleus
641	**BLM**	Bloom syndrome, RecQ helicase-like	-3.3	8.7	5.4	-1.2	7.2	6.0	Nucleus
57405	**SPC25 (incluc**	SPC25, NDC80 kinetochore complex component, homolog (S.	-3.2	9.2	5.9	-1.4	6.4	5.1	Cytoplasm
8318	**CDC45**	cell division cycle 45 homolog (S. cerevisiae)	-3.1	9.6	6.5	-1.7	8.9	7.2	Nucleus
23397	**NCAPH**	non-SMC condensin I complex, subunit H	-3.1	9.5	6.5	-0.7	8.3	7.5	Nucleus
9134	**CCNE2**	cyclin E2	-3.1	8.3	5.2	-1.4	5.6	4.2	Nucleus
54892	**NCAPG2**	non-SMC condensin II complex, subunit G2	-3.0	9.0	6.0	-0.7	7.1	6.3	Nucleus
2491	**CENPI**	centromere protein I	-3.0	8.2	5.3	-0.6	6.6	6.0	Nucleus
55388	**MCM10 (inclu**	minichromosome maintenance complex component 10	-2.9	8.5	5.6	-1.8	7.7	5.9	Nucleus
3832	**KIF11**	kinesin family member 11	-2.9	8.7	5.9	-0.5	6.4	5.9	Nucleus
7298	**TYMS**	thymidylate synthetase	-2.8	10.7	7.8	-2.4	9.0	6.7	Nucleus
675	**BRCA2**	breast cancer 2, early onset	-2.8	8.1	5.3	-0.7	6.7	6.0	Nucleus
699	**BUB1 (include**	budding uninhibited by benzimidazoles 1 homolog (yeast)	-2.8	9.2	6.5	-0.2	7.7	7.5	Nucleus
113130	**CDCA5**	cell division cycle associated 5	-2.7	9.3	6.6	-1.1	8.8	7.7	Cytoplasm
55165	**CEP55**	centrosomal protein 55kDa	-2.7	9.0	6.3	-0.1	7.1	6.9	Cytoplasm
7272	**TTK**	TTK protein kinase	-2.6	8.4	5.8	-0.1	5.9	5.8	Nucleus
26271	**FBXO5**	F-box protein 5	-2.6	9.8	7.2	-1.2	8.2	7.0	Nucleus
332	**BIRC5**	baculoviral IAP repeat containing 5	-2.6	9.8	7.2	-0.8	8.7	7.9	Cytoplasm
983	**CDK1**	cyclin-dependent kinase 1	-2.6	10.2	7.6	-0.3	7.5	7.3	Nucleus
4605	**MYBL2**	v-myb myeloblastosis viral oncogene homolog (avian)-like 2	-2.6	10.8	8.3	-1.8	9.4	7.6	Nucleus
11130	**ZWINT**	ZW10 interactor	-2.6	9.7	7.2	-1.4	8.7	7.2	Nucleus
890	**CCNA2**	cyclin A2	-2.5	9.6	7.1	-0.6	7.4	6.9	Nucleus
54443	**ANLN**	anillin, actin binding protein	-2.5	9.8	7.3	-0.5	9.1	8.5	Cytoplasm
990	**CDC6 (include**	cell division cycle 6 homolog (S. cerevisiae)	-2.5	9.4	6.9	-1.7	9.2	7.5	Nucleus
10460	**TACC3**	transforming, acidic coiled-coil containing protein 3	-2.5	9.2	6.7	-0.7	8.2	7.5	Nucleus
6502	**SKP2 (include**	S-phase kinase-associated protein 2, E3 ubiquitin protein ligas	-2.5	10.1	7.6	-1.7	8.9	7.3	Nucleus
151648	**SGOL1**	shugoshin-like 1 (S. pombe)	-2.5	8.4	5.9	-0.5	6.9	6.5	Nucleus
1033	**CDKN3**	cyclin-dependent kinase inhibitor 3	-2.4	10.5	8.1	-0.2	8.3	8.1	Nucleus
2305	**FOXM1**	forkhead box M1	-2.4	9.7	7.3	-0.8	9.4	8.6	Nucleus
991	**CDC20 (incluc**	cell division cycle 20 homolog (S. cerevisiae)	-2.4	10.1	7.7	-0.9	9.0	8.1	Nucleus
56992	**KIF15**	kinesin family member 15	-2.4	7.1	4.8	-0.4	5.5	5.2	Nucleus
55355	**HJURP**	Holliday junction recognition protein	-2.3	9.8	7.4	-0.8	8.7	7.9	Nucleus
5347	**PLK1**	polo-like kinase 1	-2.3	10.6	8.3	-0.4	8.4	8.0	Nucleus
1111	**CHEK1**	checkpoint kinase 1	-2.2	9.5	7.3	-1.5	9.4	7.9	Nucleus
220134	**SKA1**	spindle and kinetochore associated complex subunit 1	-2.2	7.4	5.1	-0.1	5.6	5.5	Nucleus
9055	**PRC1 (include**	protein regulator of cytokinesis 1	-2.2	10.5	8.3	-0.1	8.2	8.1	Nucleus
898	**CCNE1**	cyclin E1	-2.2	8.6	6.4	-1.6	8.6	7.0	Nucleus
9735	**KNTC1**	kinetochore associated 1	-2.2	8.1	5.9	-0.8	7.5	6.8	Nucleus
10592	**SMC2**	structural maintenance of chromosomes 2	-2.2	9.2	7.0	-0.5	6.7	6.2	Nucleus
10403	**NDC80**	NDC80 kinetochore complex component homolog (S. cerevisia	-2.2	8.6	6.4	-0.3	5.7	5.4	Nucleus
6491	**STIL**	SCL/TAL1 interrupting locus	-2.1	7.5	5.4	-0.1	6.4	6.3	Nucleus
9212	**AURKB**	aurora kinase B	-2.1	10.2	8.1	-0.7	8.6	7.9	Nucleus
672	**BRCA1**	breast cancer 1, early onset	-2.1	7.9	5.7	-0.7	7.0	6.3	Nucleus
8438	**RAD54L**	RAD54-like (S. cerevisiae)	-2.1	7.9	5.8	-1.0	7.5	6.5	Nucleus
5888	**RAD51**	RAD51 homolog (S. cerevisiae)	-2.1	9.1	7.0	-1.2	7.7	6.4	Nucleus
9700	**ESPL1**	extra spindle pole bodies homolog 1 (S. cerevisiae)	-2.1	8.0	5.9	-0.7	7.1	6.4	Nucleus
8317	**CDC7 (include**	cell division cycle 7 homolog (S. cerevisiae)	-2.1	7.0	5.0	-0.9	6.0	5.1	Nucleus
22974	**TPX2**	TPX2, microtubule-associated, homolog (Xenopus laevis)	-2.1	10.6	8.5	-0.2	9.2	9.0	Nucleus
23645	**PPP1R15A**	protein phosphatase 1, regulatory subunit 15A	2.0	7.0	9.0	0.9	8.4	9.4	Cytoplasm
28984	**RGCC**	regulator of cell cycle	2.3	7.9	10.2	2.2	6.9	9.0	Cytoplasm

Table S2 50 genes involved in cellular proliferation being affected upon KRT23 knockdown in SW948 cells. Data were obtained by microarray expression profiling followed by RMA normalization, comparison of SW948 control cells versus SW948-sh1506 with KRT23 knockdown (treshold log2 ratio >|2.0|). Some genes affected in SW948 were not affected or to a lesser extent affected in LS1034 cells. Proliferation marker KI67 is highlighted with grey.

			log2						
Entrez Gene ID	Symbol	description	ratio	SW948-ctrl	SW948-sh1506	ratio	LS1034-ctrl	LS1034-sh1506	Location
641	**BLM**	Bloom syndrome, RecQ helicase-like	-3.3	8.7	5.4	-1.2	7.2	6.0	Nucleus
4288	**MKI67**	antigen identified by monoclonal antibody Ki-67	-3.3	10.0	6.7	-0.9	8.5	7.6	Nucleus
8318	**CDC45**	cell division cycle 45 homolog (S. cerevisiae)	-3.1	9.6	6.5	-1.7	8.9	7.2	Nucleus
9134	**CCNE2**	cyclin E2	-3.1	8.3	5.2	-1.4	5.6	4.2	Nucleus
51514	**DTL**	denticleless E3 ubiquitin protein ligase homolog (Dros	-3.1	9.5	6.5	-1.6	9.0	7.3	Nucleus
79733	**E2F8**	E2F transcription factor 8	-2.9	8.9	6.0	-1.9	7.8	5.8	Nucleus
6241	**RRM2**	ribonucleotide reductase M2	-2.9	12.0	9.1	-1.8	11.2	9.4	Nucleus
3832	**KIF11**	kinesin family member 11	-2.9	8.7	5.9	-0.5	6.4	5.9	Nucleus
7298	**TYMS**	thymidylate synthetase	-2.8	10.7	7.8	-2.4	9.0	6.7	Nucleus
675	**BRCA2**	breast cancer 2, early onset	-2.8	8.1	5.3	-0.7	6.7	6.0	Nucleus
699	**BUB1 (incluc**	budding uninhibited by benzimidazoles 1 homolog (ye	-2.8	9.2	6.5	-0.2	7.7	7.5	Nucleus
113130	**CDCA5**	cell division cycle associated 5	-2.7	9.3	6.6	-1.1	8.8	7.7	Cytoplasm
55872	**PBK**	PDZ binding kinase	-2.6	10.2	7.6	-0.6	7.9	7.2	Cytoplasm
7272	**TTK**	TTK protein kinase	-2.6	8.4	5.8	-0.1	5.9	5.8	Nucleus
332	**BIRC5**	baculoviral IAP repeat containing 5	-2.6	9.8	7.2	-0.8	8.7	7.9	Cytoplasm
983	**CDK1**	cyclin-dependent kinase 1	-2.6	10.2	7.6	-0.3	7.5	7.3	Nucleus
4605	**MYBL2**	v-myb myeloblastosis viral oncogene homolog (avian)-	-2.6	10.8	8.3	-1.8	9.4	7.6	Nucleus
890	**CCNA2**	cyclin A2	-2.5	9.6	7.1	-0.6	7.4	6.9	Nucleus
990	**CDC6 (incluc**	cell division cycle 6 homolog (S. cerevisiae)	-2.5	9.4	6.9	-1.7	9.2	7.5	Nucleus
6502	**SKP2 (incluc**	S-phase kinase-associated protein 2, E3 ubiquitin pro	-2.5	10.1	7.6	-1.7	8.9	7.3	Nucleus
6941	**TCF19**	transcription factor 19	-2.5	9.8	7.3	-1.5	8.3	6.8	Nucleus
1033	**CDKN3**	cyclin-dependent kinase inhibitor 3	-2.4	10.5	8.1	-0.2	8.3	8.1	Nucleus
2305	**FOXM1**	forkhead box M1	-2.4	9.7	7.3	-0.8	9.4	8.6	Nucleus
56992	**KIF15**	kinesin family member 15	-2.4	7.1	4.8	-0.4	5.5	5.2	Nucleus
6839	**SUV39H1**	suppressor of variegation 3-9 homolog 1 (Drosophila)	-2.3	8.6	6.3	-0.9	7.5	6.6	Nucleus
5347	**PLK1**	polo-like kinase 1	-2.3	10.6	8.3	-0.4	8.4	8.0	Nucleus
1111	**CHEK1**	checkpoint kinase 1	-2.2	9.5	7.3	-1.5	9.4	7.9	Nucleus
9055	**PRC1 (incluc**	protein regulator of cytokinesis 1	-2.2	10.5	8.3	-0.1	8.2	8.1	Nucleus
898	**CCNE1**	cyclin E1	-2.2	8.6	6.4	-1.6	8.6	7.0	Nucleus
8914	**TIMELESS**	timeless homolog (Drosophila)	-2.2	8.8	6.6	-1.1	7.9	6.9	Nucleus
29128	**UHRF1**	ubiquitin-like with PHD and ring finger domains 1	-2.2	7.9	5.8	-2.0	7.4	5.4	Nucleus
6491	**STIL**	SCL/TAL1 interrupting locus	-2.1	7.5	5.4	-0.1	6.4	6.3	Nucleus
9212	**AURKB**	aurora kinase B	-2.1	10.2	8.1	-0.7	8.6	7.9	Nucleus
672	**BRCA1**	breast cancer 1, early onset	-2.1	7.9	5.7	-0.7	7.0	6.3	Nucleus
63967	**CLSPN**	claspin	-2.1	6.3	4.2	-0.9	5.5	4.6	Nucleus
5888	**RAD51**	RAD51 homolog (S. cerevisiae)	-2.1	9.1	7.0	-1.2	7.7	6.4	Nucleus
8317	**CDC7 (incluc**	cell division cycle 7 homolog (S. cerevisiae)	-2.1	7.0	5.0	-0.9	6.0	5.1	Nucleus
4174	**MCM5**	minichromosome maintenance complex component 5	-2.1	9.5	7.5	-1.9	9.2	7.3	Nucleus
22974	**TPX2**	TPX2, microtubule-associated, homolog (Xenopus lae\	-2.1	10.6	8.5	-0.2	9.2	9.0	Nucleus
29028	**ATAD2**	ATPase family, AAA domain containing 2	-2.1	8.6	6.5	-1.0	6.9	5.8	Nucleus
23645	**PPP1R15A**	protein phosphatase 1, regulatory subunit 15A	2.0	7.0	9.0	0.9	8.4	9.4	Cytoplasm
5376	**PMP22**	peripheral myelin protein 22	2.2	6.2	8.4	0.6	5.5	6.1	Plasma Membrane
27074	**LAMP3**	lysosomal-associated membrane protein 3	2.3	6.1	8.4	2.2	7.1	9.3	Plasma Membrane
5268	**SERPINB5**	serpin peptidase inhibitor, clade B (ovalbumin), memb	2.6	5.2	7.8	1.1	6.4	7.5	Extracellular
3606	**IL18 (include**	interleukin 18 (interferon-gamma-inducing factor)	2.6	5.5	8.1	1.8	6.6	8.4	Extracellular
1649	**DDIT3**	DNA-damage-inducible transcript 3	2.8	6.9	9.7	3.2	6.6	9.7	Nucleus
6364	**CCL20**	chemokine (C-C motif) ligand 20	3.1	5.7	8.7	2.0	7.2	9.2	Extracellular
80329	**ULBP1**	UL16 binding protein 1	3.3	4.3	7.6	2.1	5.2	7.3	Plasma Membrane
23657	**SLC7A11**	solute carrier family 7 (anionic amino acid transporter	3.5	7.0	10.4	2.3	7.7	9.9	Plasma Membrane
3576	**IL8**	interleukin 8	3.7	5.2	8.9	0.9	5.4	6.2	Extracellular

Table S3 110 genes involved in DNA replication and repair being affected upon KRT23 knockdown in SW948 cells and compared to LS1034 cells. Some genes affected in SW948 were not affected or to a lesser extent affected in LS1034 cells. Tenascin C was the only gene involved in DNA replication and repair which was strongly affected by KRT23 knockdown as well as by serum starvation in SW948 cells, it was unaffected in LS1034 cells. The effect of KRT23 knockdown on DNA repair genes was thus regarded as specific. Data were obtained by microarray expression profiling followed by RMA normalization, comparison of SW948 control cells versus SW948-sh1506 with KRT23 knockdown (treshold log2 ratio >|1.0|) followed by comparison to LS1034-ctrl and LS1034-sh-1506 KRT23 knockdown. Protein expression was analyzed in SW948-ctrl and SW948-sh1506 cells for MRE11A, E2F1, BRCA1 and RAD51 highlighted with grey.

					log2				
Entrez gene ID	Symbol	description	ratio	SW948-ctrl	SW948-sh1506	ratio	LS1034-ctrl	LS1034-sh1506	Location
54821	**ERCC6L**	excision repair cross-complementing rode	-3.6	7.9	4.2	-0.8	5.5	4.7	Cytoplasm
641	**BLM**	Bloom syndrome, RecQ helicase-like	-3.3	8.7	5.4	-1.2	7.2	6.0	Extracellular
9156	**EXO1 (include**	exonuclease 1	-3.2	9.0	5.8	-1.7	7.7	6.0	Cytoplasm
57405	**SPC25 (includ**	SPC25, NDC80 kinetochore complex com	-3.2	9.2	5.9	-1.4	6.4	5.1	Cytoplasm
8318	**CDC45**	cell division cycle 45 homolog (S. cerevis	-3.1	9.6	6.5	-1.7	8.9	7.2	Nucleus
23397	**NCAPH**	non-SMC condensin I complex, subunit H	-3.1	9.5	6.5	-0.7	8.3	7.5	Nucleus
9134	**CCNE2**	cyclin E2	-3.1	8.3	5.2	-1.4	5.6	4.2	Nucleus
54892	**NCAPG2**	non-SMC condensin II complex, subunit G	-3.0	9.0	6.0	-0.7	7.1	6.3	Nucleus
5427	**POLE2**	polymerase (DNA directed), epsilon 2 (p5	-3.0	8.0	5.0	-1.3	5.8	4.6	Nucleus
55388	**MCM10 (includ**	minichromosome maintenance complex c	-2.9	8.5	5.6	-1.8	7.7	5.9	Cytoplasm
6241	**RRM2**	ribonucleotide reductase M2	-2.9	12.0	9.1	-1.8	11.2	9.4	Nucleus
3832	**KIF11**	kinesin family member 11	-2.9	8.7	5.9	-0.5	6.4	5.9	Nucleus
675	**BRCA2**	breast cancer 2, early onset	-2.8	8.1	5.3	-0.7	6.7	6.0	Nucleus
699	**BUB1 (include**	budding uninhibited by benzimidazoles 1	-2.8	9.2	6.5	-0.2	7.7	7.5	Cytoplasm
113130	**CDCA5**	cell division cycle associated 5	-2.7	9.3	6.6	-1.1	8.8	7.7	Nucleus
55872	**PBK**	PDZ binding kinase	-2.6	10.2	7.6	-0.6	7.9	7.2	Nucleus
7272	**TTK**	TTK protein kinase	-2.6	8.4	5.8	-0.1	5.9	5.8	Nucleus
332	**BIRC5**	baculoviral IAP repeat containing 5	-2.6	9.8	7.2	-0.8	8.7	7.9	Nucleus
11130	**ZWINT**	ZW10 interactor	-2.6	9.7	7.2	-1.4	8.7	7.2	Cytoplasm
890	**CCNA2**	cyclin A2	-2.5	9.6	7.1	-0.6	7.4	6.9	Nucleus
990	**CDC6 (include**	cell division cycle 6 homolog (S. cerevisia	-2.5	9.4	6.9	-1.7	9.2	7.5	Nucleus
10460	**TACC3**	transforming, acidic coiled-coil containing	-2.5	9.2	6.7	-0.7	8.2	7.5	Nucleus
6502	**SKP2 (includes**	S-phase kinase-associated protein 2, E3	-2.5	10.1	7.6	-1.7	8.9	7.3	Cytoplasm
2305	**FOXM1**	forkhead box M1	-2.4	9.7	7.3	-0.8	9.4	8.6	Nucleus
991	**CDC20 (includ**	cell division cycle 20 homolog (S. cerevis	-2.4	10.1	7.7	-0.9	9.0	8.1	Nucleus
55355	**HJURP**	Holliday junction recognition protein	-2.3	9.8	7.4	-0.8	8.7	7.9	Nucleus
5347	**PLK1**	polo-like kinase 1	-2.3	10.6	8.3	-0.4	8.4	8.0	Nucleus
1111	**CHEK1**	checkpoint kinase 1	-2.2	9.5	7.3	-1.5	9.4	7.9	Nucleus
220134	**SKA1**	spindle and kinetochore associated comp	-2.2	7.4	5.1	-0.1	5.6	5.5	Nucleus
898	**CCNE1**	cyclin E1	-2.2	8.6	6.4	-1.6	8.6	7.0	Nucleus
9735	**KNTC1**	kinetochore associated 1	-2.2	8.1	5.9	-0.8	7.5	6.8	Cytoplasm
10592	**SMC2**	structural maintenance of chromosomes 2	-2.2	9.2	7.0	-0.5	6.7	6.2	Nucleus
8914	**TIMELESS**	timeless homolog (Drosophila)	-2.2	8.8	6.6	-1.1	7.9	6.9	Nucleus
10403	**NDC80**	NDC80 kinetochore complex component h	-2.2	8.6	6.4	-0.3	5.7	5.4	Extracellular
4173	**MCM4**	minichromosome maintenance complex c	-2.1	10.1	8.0	-1.8	10.0	8.2	Nucleus
10714	**POLD3**	polymerase (DNA-directed), delta 3, acce	-2.1	8.5	6.4	-1.1	7.9	6.9	Nucleus
**672**	**BRCA1**	**breast cancer 1, early onset**	**-2.1**	**7.9**	**5.7**	**-0.7**	**7.0**	**6.3**	**Nucleus**
8438	**RAD54L**	RAD54-like (S. cerevisiae)	-2.1	7.9	5.8	-1.0	7.5	6.5	Nucleus
**5888**	**RAD51**	**RAD51 homolog (S. cerevisiae)**	**-2.1**	**9.1**	**7.0**	**-1.2**	**7.7**	**6.4**	**Plasma Membrane**
9700	**ESPL1**	extra spindle pole bodies homolog 1 (S. c	-2.1	8.0	5.9	-0.7	7.1	6.4	Extracellular
8317	**CDC7 (include**	cell division cycle 7 homolog (S. cerevisia	-2.1	7.0	5.0	-0.9	6.0	5.1	Nucleus
4174	**MCM5**	minichromosome maintenance complex c	-2.1	9.5	7.5	-1.9	9.2	7.3	Nucleus
22974	**TPX2**	TPX2, microtubule-associated, homolog (	-2.1	10.6	8.5	-0.2	9.2	9.0	Nucleus
25788	**RAD54B**	RAD54 homolog B (S. cerevisiae)	-2.0	7.9	5.9	-0.1	6.1	6.0	Extracellular
3838	**KPNA2**	karyopherin alpha 2 (RAG cohort 1, impo	-1.9	5.8	3.8	-0.2	4.8	4.6	Nucleus
4998	**ORC1 (include**	origin recognition complex, subunit 1	-1.9	7.4	5.4	-1.5	6.8	5.3	Nucleus
10721	**POLQ**	polymerase (DNA directed), theta	-1.9	6.5	4.6	-0.7	5.8	5.1	Extracellular
4436	**MSH2**	mutS homolog 2, colon cancer, nonpolypo	-1.9	8.9	7.0	-0.9	7.3	6.4	Nucleus
5983	**RFC3**	replication factor C (activator 1) 3, 38kDa	-1.9	10.6	8.7	-1.3	9.0	7.7	Nucleus
9738	**CCP110**	centriolar coiled coil protein 110kDa	-1.9	6.5	4.6	-0.3	5.1	4.8	Cytoplasm
23310	**NCAPD3**	non-SMC condensin II complex, subunit D	-1.9	9.7	7.8	-1.2	8.9	7.8	Nucleus
2189	**FANCG**	Fanconi anemia, complementation group	-1.8	8.3	6.5	-0.8	8.1	7.3	Nucleus
10535	**RNASEH2A**	ribonuclease H2, subunit A	-1.8	10.5	8.8	-1.0	9.5	8.5	Nucleus
993	**CDC25A**	cell division cycle 25 homolog A (S. pomb	-1.8	8.3	6.5	-1.0	8.0	7.0	Nucleus
4175	**MCM6**	minichromosome maintenance complex c	-1.7	9.7	8.0	-1.4	8.8	7.5	Nucleus
3835	**KIF22**	kinesin family member 22	-1.7	9.4	7.7	-0.9	8.4	7.6	Nucleus
5933	**RBL1**	retinoblastoma-like 1 (p107)	-1.6	8.7	7.1	-1.3	8.3	7.0	Nucleus
55706	**TMEM48**	transmembrane protein 48	-1.6	8.8	7.2	-0.8	7.7	6.9	Nucleus
51053	**GMNN**	geminin, DNA replication inhibitor	-1.6	9.7	8.2	-1.1	8.5	7.3	Nucleus
23594	**ORC6 (include**	origin recognition complex, subunit 6	-1.6	8.7	7.1	-0.8	7.9	7.1	Nucleus
1643	**DDB2**	damage-specific DNA binding protein 2, 4	-1.5	8.9	7.4	-0.6	8.9	8.3	Nucleus

Table S3 is continued on next page

Table S3 - continued from previous page

Entrez gene ID	Symbol	description	log2 ratio	SW948-ctrl	SW948-sh1506	ratio	LS1034-ctrl	LS1034-sh1506	Location
3619	**INCENP**	inner centromere protein antigens 135/155kD	-1.5	9.0	7.5	-0.7	8.4	7.8	Nucleus
4171	**MCM2**	minichromosome maintenance complex comp	-1.5	9.5	8.0	-1.5	9.5	8.1	Nucleus
3978	**LIG1**	ligase I, DNA, ATP-dependent	-1.5	8.4	6.9	-1.1	8.0	6.9	Nucleus
5426	**POLE**	polymerase (DNA directed), epsilon	-1.5	8.2	6.7	-1.1	8.6	7.6	Nucleus
55038	**CDCA4**	cell division cycle associated 4	-1.5	10.2	8.8	-1.0	9.3	8.3	Nucleus
1786	**DNMT1**	DNA (cytosine-5-)-methyltransferase 1	-1.5	9.5	8.0	-0.9	9.0	8.1	Nucleus
4172	**MCM3**	minichromosome maintenance complex comp	-1.5	9.5	8.0	-1.3	8.8	7.5	Nucleus
79980	**DSN1 (includes**	DSN1, MIND kinetochore complex componen	-1.5	9.6	8.2	-0.6	8.3	7.7	Nucleus
2030	**SLC29A1**	solute carrier family 29 (nucleoside transporte	-1.5	10.5	9.0	-0.8	9.5	8.7	Nucleus
7112	**TMPO**	thymopoietin	-1.5	10.2	8.7	-0.8	8.7	7.9	Nucleus
146956	**EME1**	essential meiotic endonuclease 1 homolog 1 (	-1.4	6.9	5.5	-0.4	6.4	6.0	Cytoplasm
5558	**PRIM2**	primase, DNA, polypeptide 2 (58kDa)	-1.4	7.9	6.5	-0.4	5.7	5.3	Nucleus
9131	**AIFM1**	apoptosis-inducing factor, mitochondrion-ass	-1.4	8.7	7.4	-0.1	7.4	7.3	Nucleus
896	**CCND3**	cyclin D3	-1.4	9.3	7.9	-0.7	8.3	7.7	Nucleus
8243	**SMC1A**	structural maintenance of chromosomes 1A	-1.4	9.6	8.2	-0.7	8.6	7.9	Nucleus
5982	**RFC2**	replication factor C (activator 1) 2, 40kDa	-1.3	10.0	8.7	-1.1	9.0	7.9	Nucleus
81620	**CDT1**	chromatin licensing and DNA replication factc	-1.3	9.0	7.7	-1.5	9.0	7.4	Nucleus
11144	**DMC1**	DMC1 dosage suppressor of mck1 homolog,	-1.3	4.9	3.6	-0.1	3.2	3.0	Nucleus
23468	**CBX5**	chromobox homolog 5	-1.3	10.2	8.9	-0.8	9.0	8.2	Nucleus
9401	**RECQL4**	RecQ protein-like 4	-1.3	8.5	7.3	-1.1	8.3	7.2	Cytoplasm
142	**PARP1**	poly (ADP-ribose) polymerase 1	-1.3	8.6	7.3	-0.1	7.6	7.4	Nucleus
5429	**POLH**	polymerase (DNA directed), eta	-1.3	8.2	6.9	-0.5	6.7	6.1	Nucleus
**4361**	**MRE11A**	**MRE11 meiotic recombination 11 homolog**	**-1.3**	**6.5**	**5.3**	**-0.5**	**5.9**	**5.5**	**Nucleus**
11200	**CHEK2**	checkpoint kinase 2	-1.2	6.9	5.7	0.0	5.3	5.3	Nucleus
5111	**PCNA**	proliferating cell nuclear antigen	-1.2	11.5	10.4	-1.6	11.0	9.4	Nucleus
3014	**H2AFX**	H2A histone family, member X	-1.2	9.8	8.7	-0.8	9.5	8.7	Nucleus
4085	**MAD2L1**	MAD2 mitotic arrest deficient-like 1 (yeast)	-1.2	10.2	9.1	-0.8	8.5	7.7	Nucleus
1017	**CDK2**	cyclin-dependent kinase 2	-1.1	9.8	8.6	-0.5	8.5	8.0	unknown
91419	**XRCC6BP1**	XRCC6 binding protein 1	-1.1	7.7	6.5	-0.1	5.9	5.8	Nucleus
2176	**FANCC**	Fanconi anemia, complementation group C	-1.1	8.1	6.9	-0.6	6.8	6.2	Nucleus
7517	**XRCC3**	X-ray repair complementing defective repair i	-1.1	8.0	6.9	-0.9	7.5	6.6	Cytoplasm
6117	**RPA1**	replication protein A1, 70kDa	-1.1	9.4	8.3	-0.2	8.0	7.8	Plasma Membr
11073	**TOPBP1**	topoisomerase (DNA) II binding protein 1	-1.1	9.0	7.9	-0.3	8.4	8.1	Nucleus
**1869**	**E2F1**	**E2F transcription factor 1**	**-1.1**	**8.6**	**7.5**	**-1.6**	**9.4**	**7.8**	**Nucleus**
57122	**NUP107**	nucleoporin 107kDa	-1.0	9.4	8.3	-0.4	8.3	7.9	Nucleus
5424	**POLD1**	polymerase (DNA directed), delta 1, catalytic	-1.0	8.1	7.0	-1.0	8.2	7.1	Nucleus
55775	**TDP1 (includes**	tyrosyl-DNA phosphodiesterase 1	-1.0	9.0	8.0	-0.1	7.8	7.7	Nucleus
5591	**PRKDC**	protein kinase, DNA-activated, catalytic polyp	-1.0	9.2	8.2	-1.2	9.5	8.3	Nucleus
56852	**RAD18**	RAD18 homolog (S. cerevisiae)	-1.0	8.9	7.9	-0.5	7.6	7.1	Nucleus
1647	**GADD45A**	growth arrest and DNA-damage-inducible, alp	1.0	8.7	9.7	1.3	8.1	9.3	Nucleus
3552	**IL1A**	interleukin 1, alpha	1.1	3.4	4.5	0.2	4.2	4.4	Nucleus
8780	**RIOK3**	RIO kinase 3 (yeast)	1.1	8.7	9.8	1.2	8.1	9.3	Extracellular
374	**AREG/AREGB**	amphiregulin	1.1	10.3	11.4	0.4	11.2	11.6	Nucleus
7422	**VEGFA**	vascular endothelial growth factor A	1.1	8.9	10.0	1.8	7.6	9.4	Nucleus
6385	**SDC4**	syndecan 4	1.2	9.4	10.6	0.3	10.6	10.9	Nucleus
1839	**HBEGF**	heparin-binding EGF-like growth factor	1.6	7.3	8.9	1.0	7.6	8.6	Nucleus
2069	**EREG**	epiregulin	1.8	7.7	9.5	0.6	10.2	10.9	Extracellular
23645	**PPP1R15A**	protein phosphatase 1, regulatory subunit 15A	2.0	7.0	9.0	0.9	8.4	9.4	Nucleus
1649	**DDIT3**	DNA-damage-inducible transcript 3	2.8	6.9	9.7	3.2	6.6	9.7	unknown

SUPPLEMENTARY FIGURE LEGENDS

Figure S1 Location of Bead Arrays probes and in vitro demethylation. A) Location of Illumina Bead array probes within chr17:39,077,462-39,099,789 including the KRT23 promotor; the interrogated CG-sites at position cg06378617 and cg22392708 are marked red. Position cg22392708 corresponding to position 116 of the fragment referred to in Figure 2. B) RT-qPCR analysis of DLD1 cells and HCT116 treated with 5'-AZA-dC showed that KRT23 transcript increased dramatically upon demethylation of the KRT23 promotor by AZA treatment.

Figure S2 Knockdown efficiency. Stable lentiviral mediated knockdown of KRT23 in different human colon cancer cell lines using five different sh-RNA constructs. A) SW480 cells stably transfected with five different sh-RNA sequences targeting KRT23 were analyzed by RTqPCR normalized to GAPD, PPIA, HPRT or B2M. The most efficient constructs were sh-1506 and sh-1010. B) LS1034 cells stably transfected with sh-1506 targeting KRT23 analyzed by RTqPCR. C) SW948 cells stably transfected with sh-1506 targeting KRT23 analyzed by RTqPCR. D) SW948 cells stably transfected with sh-1506 analyzed by RTqPCR using a Taqman probe, data were normalized to UBC. E) Microarray expression profiling of SW480, SW948 and LS1034 MSS colon cancer cell lines, either a control with an empty vector or a stable knockdown using the sh1506 construct. Data were normalized by two different normalization strategies using RMA normalization (Robust Multichip Average) or the iterPLIER expression console (Probe Logarithmic Intensity Error). iterPLIER may focus on single, highly expressed probesets with high GC content (hybridization) which may not be representative and thus we refer to RMA normalized data in the main text.

Figure S3 KRT23 depletion also affects proliferation of LS1034 and SW480 cells. All experiments described in the main text performed on SW948 derived cell lines (SW948-ctrl, SW948-sh1506 and SW948-sh1010) were also performed with LS1034-ctrl, LS1034-sh1506, LS1034-sh1010, SW480-ctrl and SW480-sh1506 cell lines. **A)-C) LS1034 cells.** A) LS1034-ctrl and –sh1506 cells were seeded with 8000 cells / well on 16-well RTCA-plates. Experiments were performed in triplicates, values are shown as medians and standard deviations for each group at selected times for a representative experiment. Proliferation of LS1034 cells with a stable KRT23 knockdown was remarkably decreased at the beginning. B) LS1034-ctrl and –sh1506 cells were seeded with 8000 cells per well (n=10) on 96-well plates and proliferation was analyzed 48h post-seeding using a MTT assay. Proliferation of KRT23 depleted cells was significantly decreased (p=9E-16). C) Visual inspection of LS1034-ctrl cells versus LS1034-sh1506 or LS1034-sh1010 cells stably transfected with different knockdown constructs indicated a lower cell density for the cells with a stable KRT23 knockdown. **D)-F) SW480 cells.** A)SW480-sh1506 colon cancer cells

depleted of KRT23 (●) and SW948-ctrl with an empty vector (○) were seeded on 96-well or 16-well RTCA-plates. Experiments were performed in triplicates, values are shown as medians and standard deviations for each group at selected times for a representative experiment. Results obtained by RTCA were corroborated by conventional MTT assay. B) SW480 cells were seeded with 8000 cells per well (n=10) on 96-well plates and proliferation was analyzed 48h post-seeding using a MTT assay. Proliferation of KRT23 depleted cells was significantly decreased (p=6.8E-04). C) Immunofluorescence analysis showed that KRT23 depleted SW480 cells showed less nuclear expression of the proliferation marker KI67 compared to control cells (Alexa 488, green), nuclei were stained with Hoechst 33342 (blue), magnification 630x**.**

Figure S4. SW948 and LS1034 control cells respond to serum withdrawal. Viability was measured by a MTT assay 24h (A) or 48 h (B) after serum-withdrawal. Media contained 10% or 0% serum, respectively.

Supplementary Figure S1

A

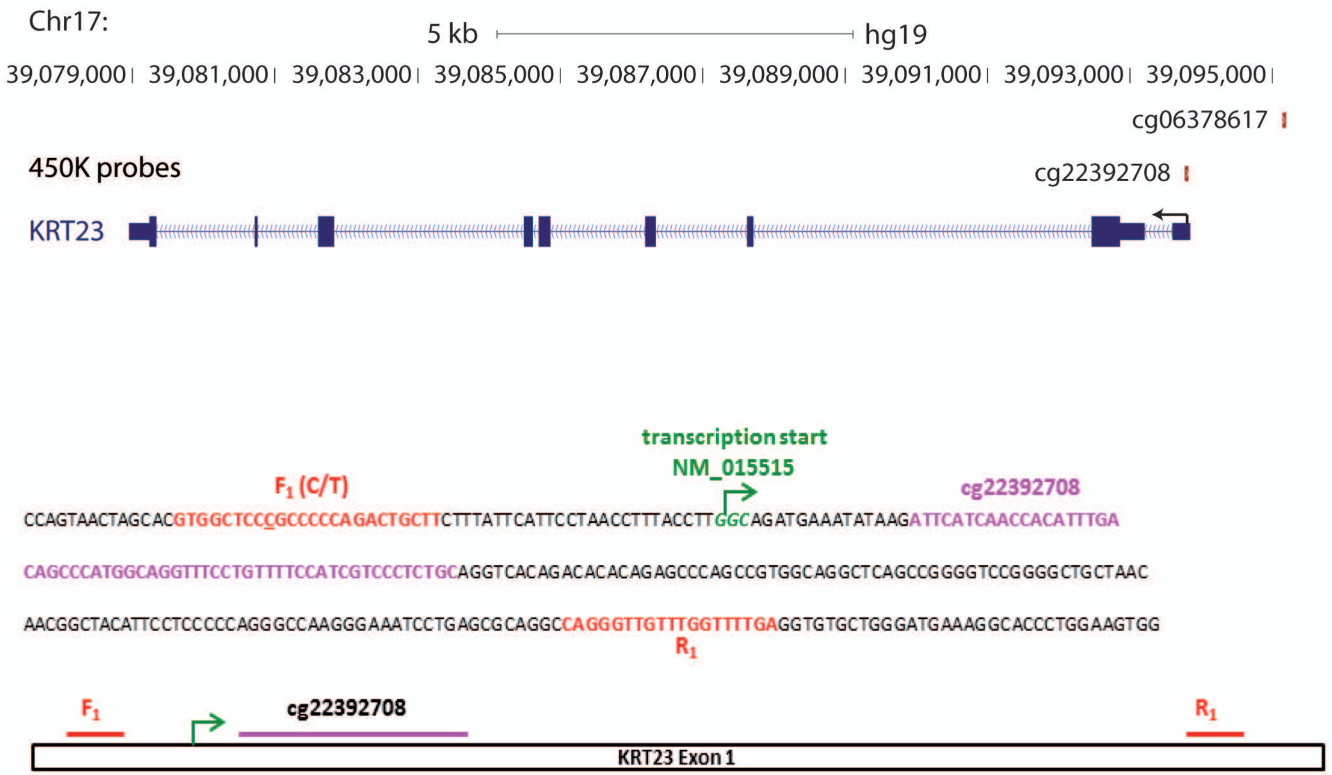

B

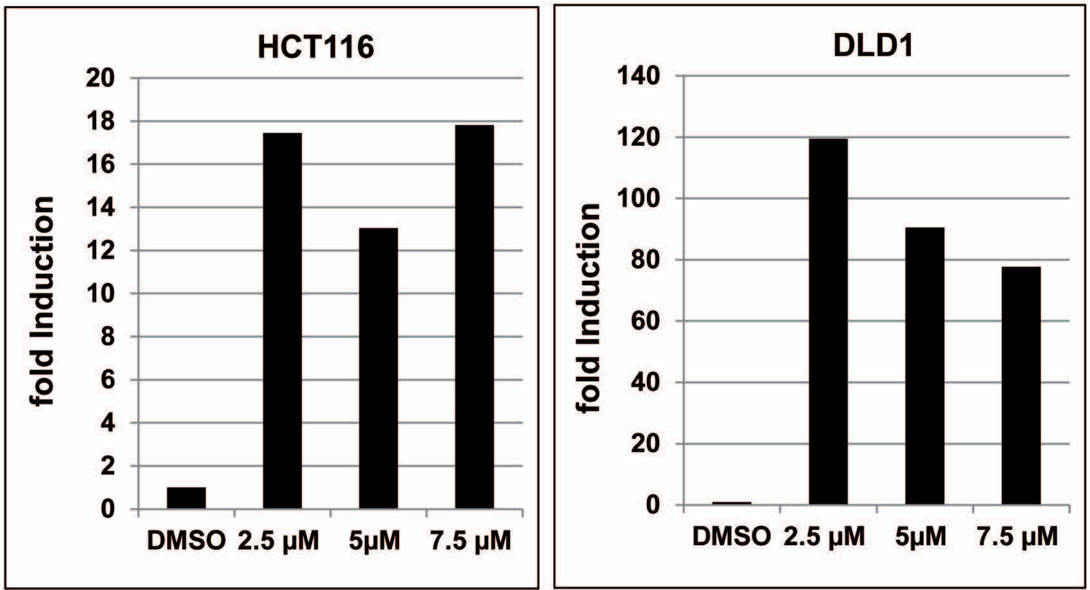

Supplementary Figure S2

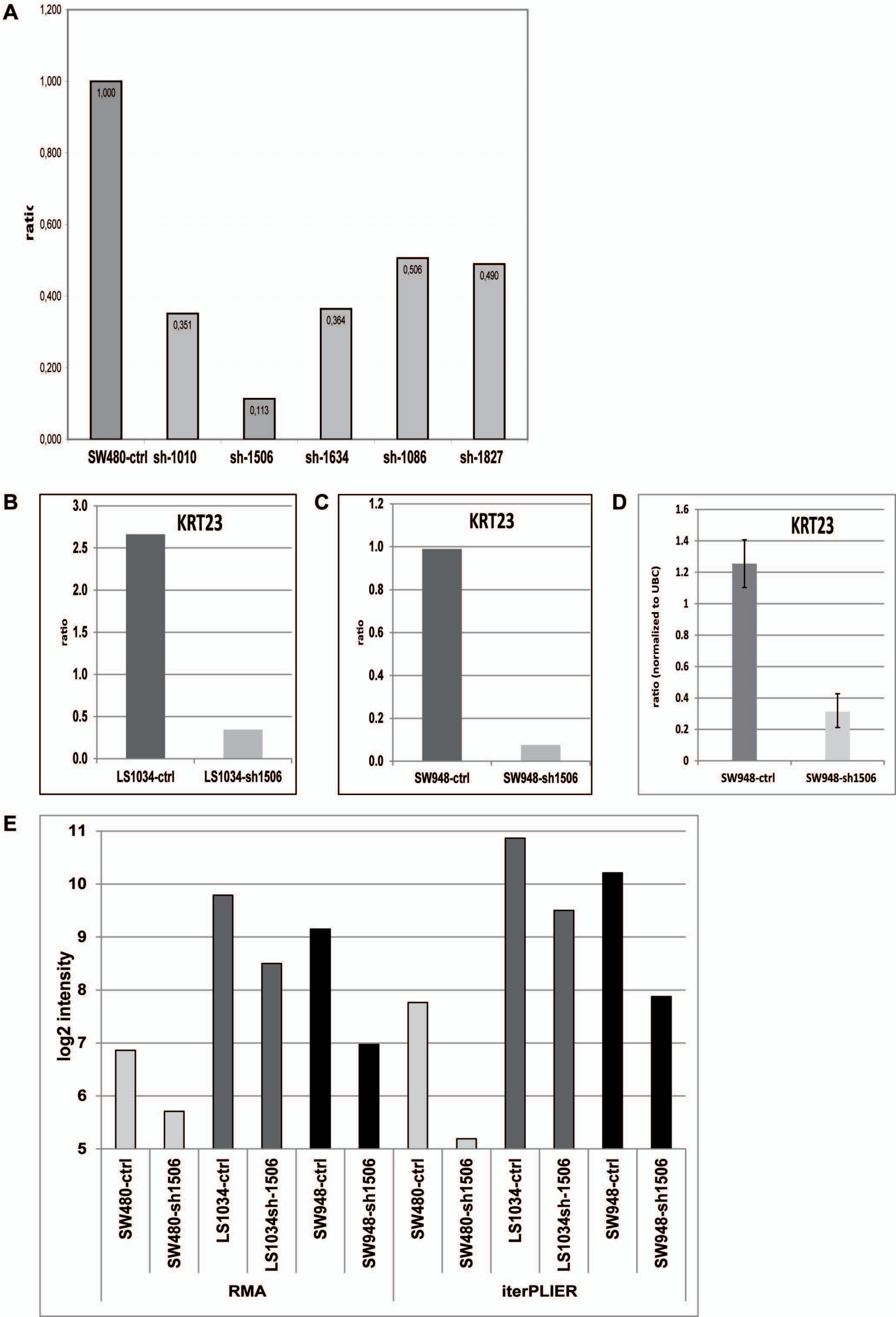

Supplementary Figure S3

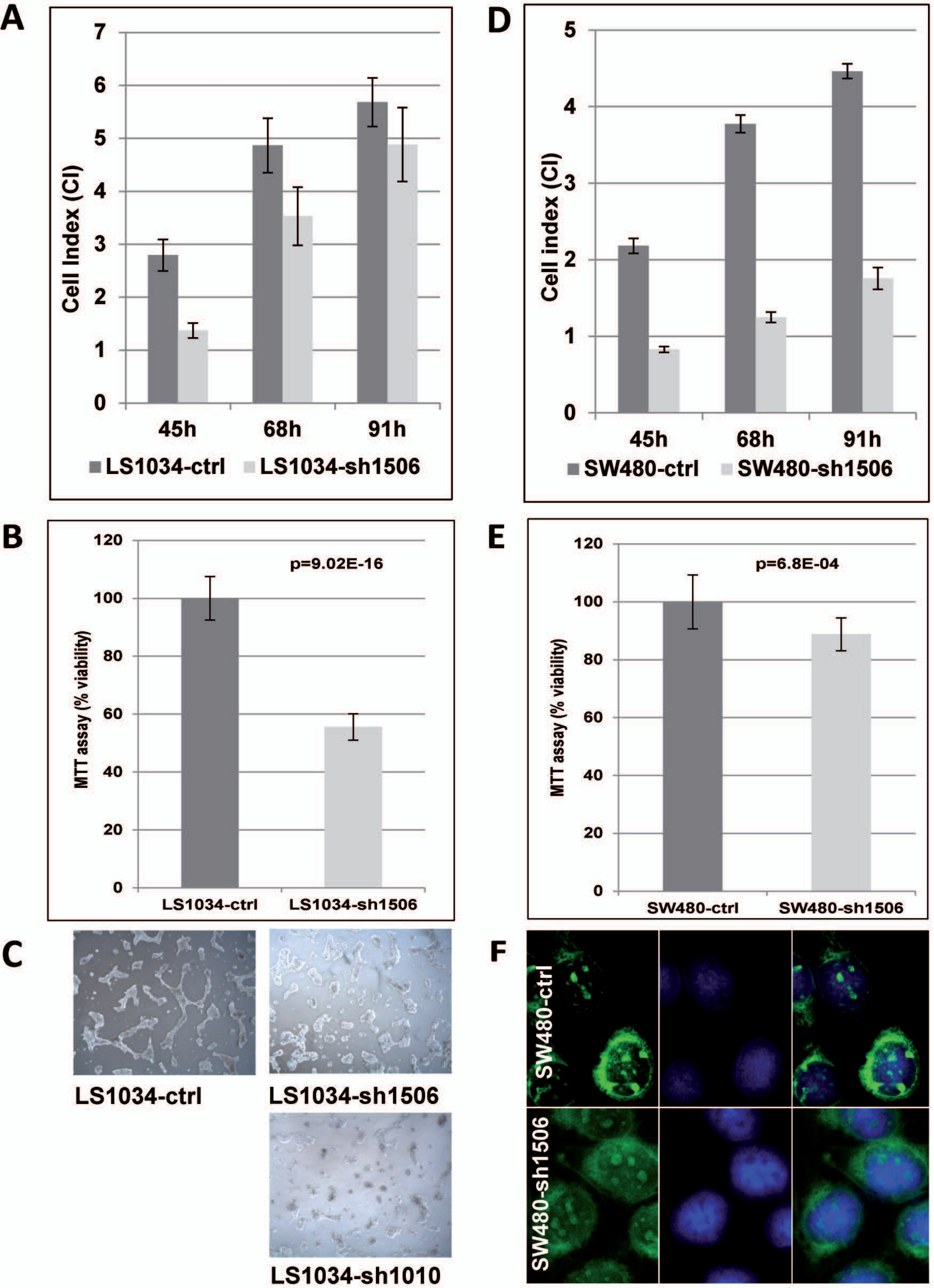

Supplementary Figure S4

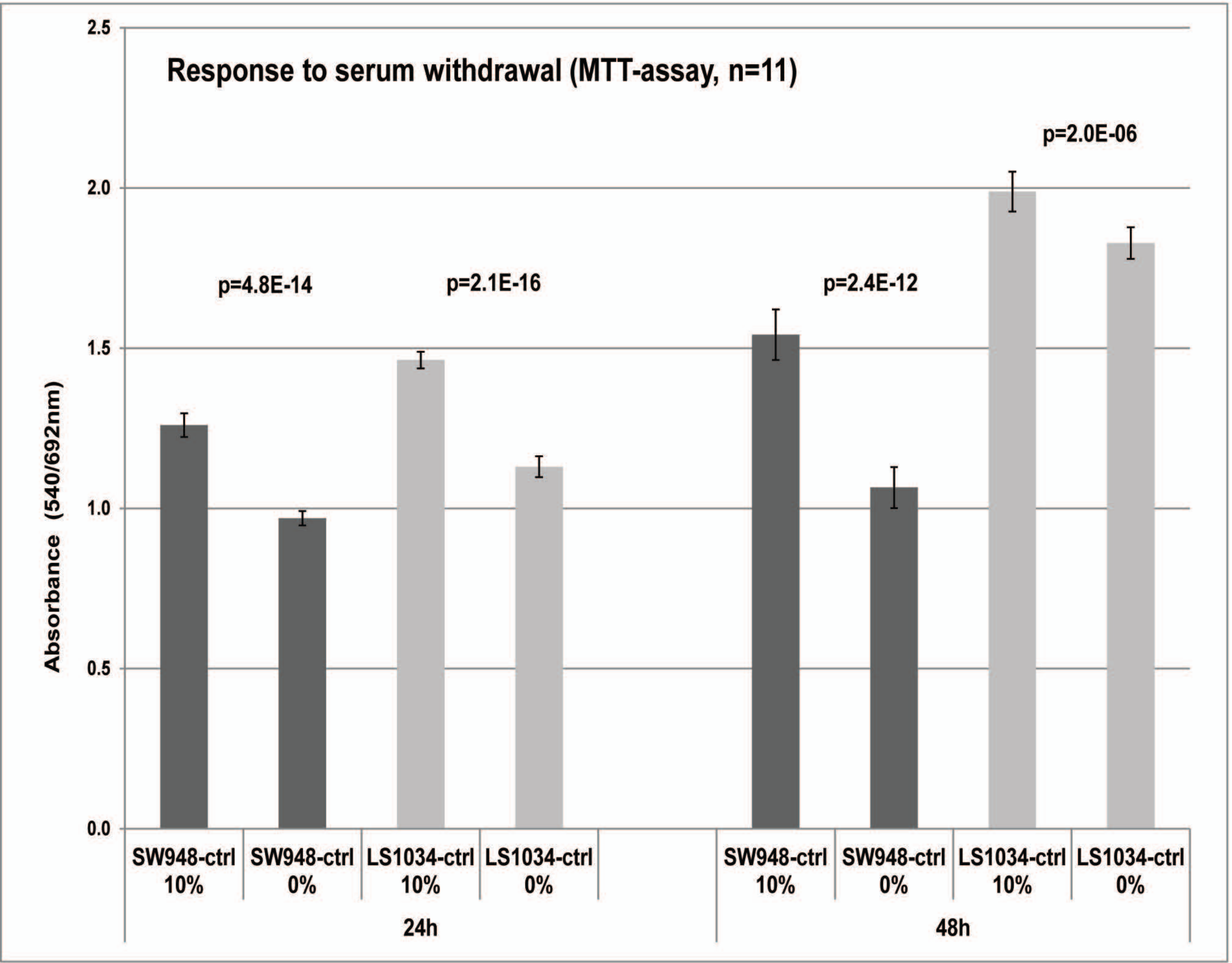

Supplement: File S1 — A combined supporting information file containing supplementary data, three supplementary Tables S1–S3, supplementary figure legends and four supplementary Figures S1–S4. (PDF) [file pone.0073593.s001.pdf]
